# Genetic Evidence for Causal Effects of Domain-Specific Physical Activity and Sedentary Behavior on Osteoporosis and Inflammatory Arthritis: A Bidirectional Mendelian Randomization Study

**DOI:** 10.3390/genes17080939

**Published:** 2026-08-12

**Authors:** Tianyu Sun, Feiyao Zhang, Chang Liu, Junhao Huang

**Affiliations:** 1 Guangdong Provincial Key Laboratory of Physical Activity and Health Promotion, Guangzhou Sport University, 1268 Middle Guangzhou Avenue, Guangzhou 510500, China; 105852024100048@stu.gzsport.edu.cn (T.S.); 19899296938@163.com (F.Z.); 2 School of Medicine, Nankai University, Tianjin 300071, China; changliu@nankai.edu.cn

**Keywords:** physical activity, sedentary behavior, Mendelian randomization, genome-wide association study, psoriatic arthritis, genetic epidemiology

## Abstract

Objective: Genetic factors contribute to physical activity (PA) and sedentary behavior (SB), two complex behavioral traits. They may be associated with musculoskeletal and inflammatory diseases. However, the relationships of domain-specific PA and SB traits with osteoporosis (OS), psoriatic arthritis (PsA), and rheumatoid arthritis (RA) remain unclear. A two-sample Mendelian randomization (MR) analysis assessed potential bidirectional associations between these behavioral traits and the three diseases. Methods: GWAS summary statistics were used to examine five domain-specific PA traits, three SB traits, and OS, RA, and PsA. Primary causal estimates were obtained using the inverse-variance weighted (IVW) method. Complementary analyses used MR-Egger regression, the weighted median method, and the weighted mode method. Sensitivity analyses were performed using Cochran’s Q test, the MR-Egger intercept test, MR-PRESSO, and leave-one-out analysis. Multiple testing was addressed using false discovery rate (FDR) correction. Results: In the forward MR analyses, genetically predicted liability to Light DIY was associated with lower odds of PsA after FDR correction (OR = 0.006, 95% CI = 0.0002–0.236, FDR-adjusted *p* = 0.018). Genetically predicted longer television viewing was linked to increased odds of PsA (OR = 2.205, 95% CI = 1.089–4.463, FDR-adjusted *p* = 0.028) and RA (OR = 1.006, 95% CI = 1.002–1.010, FDR-adjusted *p* = 0.004). Walking for pleasure showed a nominal inverse association with PsA, and computer use showed a nominal inverse association with RA. Neither association remained significant after FDR correction. No evidence of associations with the broad self-reported OS diagnosis was observed. Reverse MR analyses showed lower odds of Walking for pleasure (OR = 0.662, 95% CI = 0.484–0.906, FDR-adjusted *p* = 0.013) and Other exercises (OR = 0.651, 95% CI = 0.490–0.864, FDR-adjusted *p* = 0.006) for genetic liability to RA. RA liability was also associated with longer television viewing time (β = 0.708, 95% CI = 0.251–1.165, FDR-adjusted *p* = 0.006). No reverse associations were observed for PsA. Conclusions: This bidirectional MR study identified several potential genetically predicted associations of domain-specific PA and SB traits with PsA and RA. The findings for television viewing and RA may suggest a potential bidirectional association. However, the results should be considered hypothesis-generating. These findings require independent replication and further validation before causal or clinical conclusions can be drawn.

## 1. Introduction

Common musculoskeletal and inflammatory disorders include osteoporosis (OS), psoriatic arthritis (PsA), and rheumatoid arthritis (RA). These disorders are associated with pain, functional impairment, disability, and reduced quality of life. Although these diseases differ in etiology, pathogenesis, and clinical presentation, they impose substantial health burdens. Lifestyle-related behavioral factors, particularly physical activity (PA) and sedentary behavior (SB), may contribute to their development and progression [1,2,3]. OS affects approximately 500 million people worldwide, and its prevalence continues to rise with population aging [4]. The disease also imposes a considerable socioeconomic burden. In the United States, the annual economic cost of OS is projected to reach $25.3 billion [5]. RA affects approximately 1% of the global population and is associated with increased mortality and poorer quality of life [6,7]. PsA is a chronic inflammatory disease commonly affecting individuals with psoriasis. It affects approximately 0.1% to 1% of the global population, and 20–30% of patients with psoriasis eventually develop PsA [8]. Unlike RA, PsA is characterized by both bone destruction and pathological new bone formation, reflecting its distinct pattern of skeletal involvement [9]. Together, these disorders highlight the need for more effective strategies for disease prevention and risk stratification.

Extensive research has examined PA as a modifiable behavioral factor in the prevention and management of OS, RA, and PsA. Randomized controlled trials have shown that PA can improve clinical outcomes and reduce disease burden in these conditions [10,11]. A meta-analysis of 97 trials found that aerobic exercise, resistance training, and combined exercise increased bone mineral density (BMD) in patients with OS and osteopenia [12]. Exercise also reduced pain, improved joint mobility, increased muscle strength, and enhanced cardiovascular function in patients with RA, with minimal adverse effects [13]. Comparable benefits have been observed in patients with PsA. Exercise interventions improved physical function, reduced pain, and enhanced quality of life [11].

Unlike PA, SB has gained increasing attention as an independent behavioral risk factor for adverse health outcomes. Common sedentary activities include television viewing, computer use, and driving. SB is defined as any waking behavior with an energy expenditure of ≤1.5 metabolic equivalents (METs) while in a sitting, reclining, or lying posture [14,15]. SB is associated with an increased risk of multiple chronic diseases, including cardiovascular and musculoskeletal disorders [16]. Previous studies have suggested that prolonged sitting reduces mechanical loading on bone tissue and may impair bone remodeling, potentially increasing the risk of fragility fractures [17]. Evidence directly linking SB to RA remains limited. However, SB has been associated with elevated systemic inflammatory markers that may contribute to the development and progression of RA [18]. Patients with PsA and higher SB levels have also shown elevated chronic low-grade inflammatory markers, including C-reactive protein and interleukin-6. Chronic inflammation is a key driver of PsA pathogenesis and may partly explain the association between SB and PsA [19].

Although previous studies have reported associations between PA, SB, and the risks of OS, RA, and PsA, most evidence comes from observational studies. Consequently, residual confounding and reverse causation may influence these associations and limit causal inference. Mendelian randomization (MR) provides a genetic epidemiological framework for causal investigation. Genetic variants serve as instrumental variables (IVs) to assess potential causal relationships between exposures and outcomes. The method has been widely used in epidemiology, genetics, and public health research to evaluate the potential effects of modifiable exposures. Random allocation of genetic variants occurs at conception, and these variants remain stable throughout life. Consequently, MR is less vulnerable to confounding and reverse causation than conventional observational studies. This framework is conceptually similar to the randomization process used in randomized controlled trials [20]. Conventional MR assesses a potential exposure-to-outcome effect. Bidirectional MR evaluates potential effects in both directions and enables assessment of causal direction and potential reverse causality. This study used bidirectional two-sample MR to assess potential causal relationships of five domain-specific PA traits and three SB traits with OS, RA, and PsA. Reverse causality was also evaluated.

## 2. Methods

### 2.1. Study Design

**Figure 1:**
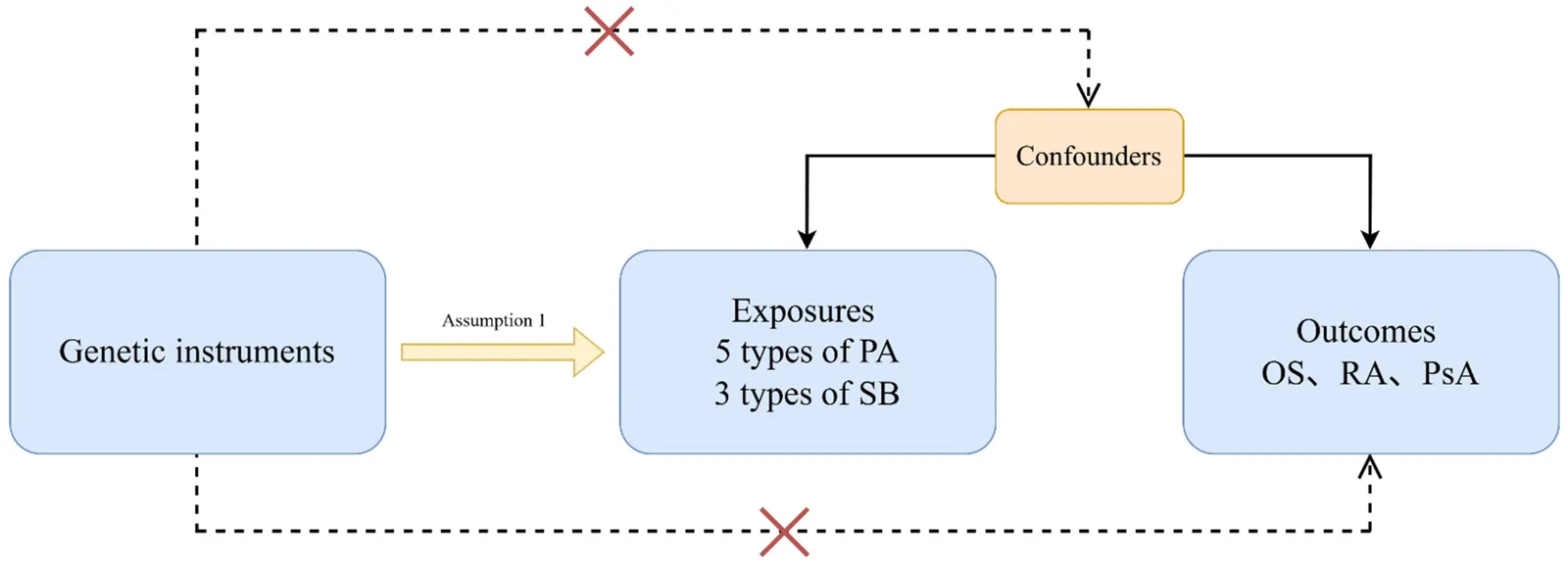
Overview of the study design and analytical workflow of the bidirectional Mendelian randomization analyses. Dashed arrows with red crosses represent the exclusion of associations between genetic instruments and confounders and of direct pathways from genetic instruments to outcomes. PA: physical activity; SB: sedentary behavior; OS: osteoporosis; RA: rheumatoid arthritis; PsA: psoriatic arthritis.

This study investigated the potential causal relationships between PA/SB traits and OS, RA, and PsA. Publicly available genome-wide association studies (GWAS) summary statistics provided the data for the two-sample MR analysis. Each original GWAS had received ethical approval, and all participants had provided informed consent. Genetic IVs were selected based on the three core assumptions of MR [21,22]: (1) the IVs were strongly associated with the exposure traits; (2) the IVs were independent of potential confounders; and (3) the IVs influenced the outcomes only through the exposure traits, not through alternative pathways. Effect allele alignment was performed across the exposure and outcome datasets. To minimize strand ambiguity, palindromic single-nucleotide polymorphisms (SNPs, A/T or C/G) were handled conservatively. SNPs without allele frequency information were excluded. SNPs with a minor allele frequency (MAF) ranging from 0.42 to 0.58 were also removed. Figure 1 presents the overall study design.

### 2.2. Data Sources and Instrumental Variable Selection

Five PA traits (Heavy DIY, Light DIY, Strenuous sports, Walking for pleasure, and Other exercises) and three SB traits (Time spent watching television, Time spent using a computer, and Time spent driving) were included in the analysis. Large-scale GWAS provided the genetic association data for these traits.

The UK Biobank provided GWAS summary statistics for the PA and SB traits. This large prospective cohort includes over 500,000 participants aged 40–69 years. The UK Biobank provides extensive phenotypic and genotypic data collected via questionnaires, physical measurements, biological sample analyses, accelerometry, imaging assessments, and genome-wide genotyping [23]. GWAS summary statistics for PA were generated from 460,370 UK Biobank participants and included 9,851,867 SNPs based on International Physical Activity Questionnaire (IPAQ) assessments [24]. The analyzed PA traits were Heavy DIY (e.g., weeding, lawn mowing, carpentry, and digging), Light DIY (e.g., pruning and watering the lawn), Strenuous sports, Walking for pleasure (not as a means of transport), and Other exercises (e.g., swimming, cycling, fitness activities, and bowling). In the original UK Biobank GWAS, these PA traits were defined based on participants’ self-reported participation in specific activities during the previous 4 weeks and were analyzed as binary traits (yes/no). Therefore, the MR estimates represent the effect of genetically predicted liability to participation in each PA trait on the risk of OS, RA, and PsA. An OR < 1 indicates that a higher genetically predicted propensity for participation in the corresponding PA trait is associated with a lower risk of the outcome. In contrast, an OR above 1 reflects a higher risk of the outcome. For each PA trait, SNPs were selected at *p* < 5 × 10^−8^. This cutoff represents the genome-wide significance threshold. Independent variants were retained after linkage disequilibrium (LD) clumping with r^2^ = 0.001 and a 10,000 kb window.

SB-related GWAS summary statistics were obtained from a GWAS of 437,887 UK Biobank participants and included 9,851,867 SNPs [25]. Three SB traits were defined based on self-reported daily time spent watching television, driving, and using a computer. Participants reported the typical daily duration of each activity, with a value of 0 indicating no time spent on that activity. In the original GWAS, these SB traits were standardized before genetic association analysis. Therefore, the MR estimates quantify changes in the risk of OS, RA, and PsA corresponding to a genetically predicted 1-standard-deviation (SD) increase in each SB trait. An OR > 1 indicates that higher genetically predicted SB levels are associated with an increased risk of the outcome. By contrast, an OR below 1 reflects a lower risk of the outcome.

GWAS summary data for RA and OS came from previously published datasets reported by Handan Melike Dönertaş et al. The RA dataset included 5427 cases and 479,171 controls. The OS dataset included 7751 cases and 476,847 controls. The FinnGen database (https://www.finngen.fi/en, accessed on 15 April 2025) provided GWAS summary statistics for PsA, with 1553 cases and 147,221 controls. Detailed information on the outcome GWAS datasets, including GWAS IDs, phenotype definitions, diagnostic criteria/coding, covariates, and ancestry information, is provided in Supplementary Table S13 [26].

### 2.3. Statistical Analyses

R software (version 4.3.3) was used for all statistical analyses. Data extraction, harmonization, and MR analysis were carried out with the TwoSampleMR package (version 0.6.8). The extract_instruments() function was used to extract genetic instruments for the exposure traits. Corresponding SNP associations in the outcome GWAS datasets were retrieved with the extract_outcome_data() function. The resulting dataset contained effect alleles, other alleles, allele frequencies, effect estimates, standard errors, and *p* values. Effect alleles across the exposure and outcome datasets were aligned with the harmonise_data() function. SNPs with incompatible alleles or ambiguous strand orientation were excluded. The harmonized datasets were then used for MR analyses.

Causal estimates were primarily obtained using the inverse-variance weighted (IVW) method [27], with MR-Egger regression [28], weighted median [29], and weighted mode methods [30] used as complementary approaches. These analyses were performed using the mr() function in the TwoSampleMR package. The IVW method combines SNP-specific estimates using inverse-variance weights. MR-Egger regression accounts for directional horizontal pleiotropy. The weighted median method uses the median of the weighted SNP-specific estimates. The weighted mode method uses the most common value among the SNP-specific estimates.

IVs must satisfy the core MR assumptions to provide valid causal estimates. Violations of these assumptions may introduce bias and affect the reliability of MR results. Horizontal pleiotropy refers to effects of genetic variants on the outcome through pathways independent of the exposure. This violates the exclusion restriction assumption of MR [31]. Sensitivity analyses were conducted to assess the robustness of the MR findings. Heterogeneity among SNP-specific estimates was examined with Cochran’s Q test [27]. A corresponding *p* value < 0.05 indicated significant heterogeneity. Assessment of horizontal pleiotropy employed the MR-Egger intercept test and MR-PRESSO analysis. The MR-Egger intercept test was carried out with the mr_pleiotropy_test() function in the TwoSampleMR package. MR-PRESSO was conducted using the MR-PRESSO package (version 1.0) to identify potential outlier variants and evaluate horizontal pleiotropy. The strength of the IVs for PA/SB traits was assessed using the F-statistic. The F-statistic was calculated as F = (β/SE)^2^, where β represents the SNP-exposure effect estimate and SE represents the corresponding standard error [32]. An F-statistic > 10 was considered evidence of sufficient instrument strength. Values below 10 indicated possible weak instrument bias. Selected IVs were further evaluated using PhenoScanner [33] (https://www.repository.cam.ac.uk/handle/1810/293487, accessed on 15 April 2025) to identify associations with potential confounding traits. Variants associated with potential confounders were excluded before analysis. Leave-one-out analysis was conducted using the mr_leaveoneout() function to determine whether individual SNPs had a disproportionate influence on IVW causal estimates. False discovery rate (FDR) correction was applied to *p* values from all MR methods to address multiple testing. Statistical significance was defined as an FDR-adjusted *p* value below 0.05.

Reverse MR analysis was performed to assess potential reverse causality. OS, RA, and PsA were treated as exposures. PA and SB traits were treated as outcomes. Effect estimates quantified the change in each outcome corresponding to a one-unit increase in the log odds of genetic liability to the disease. For binary PA outcomes, the MR estimates were exponentiated and reported as ORs with 95% CIs. For standardized continuous SB outcomes, the original MR estimates were reported as β coefficients with 95% CIs. An OR of 1 and a β coefficient of 0 were considered the null values.

## 3. Results

**Figure 2:**
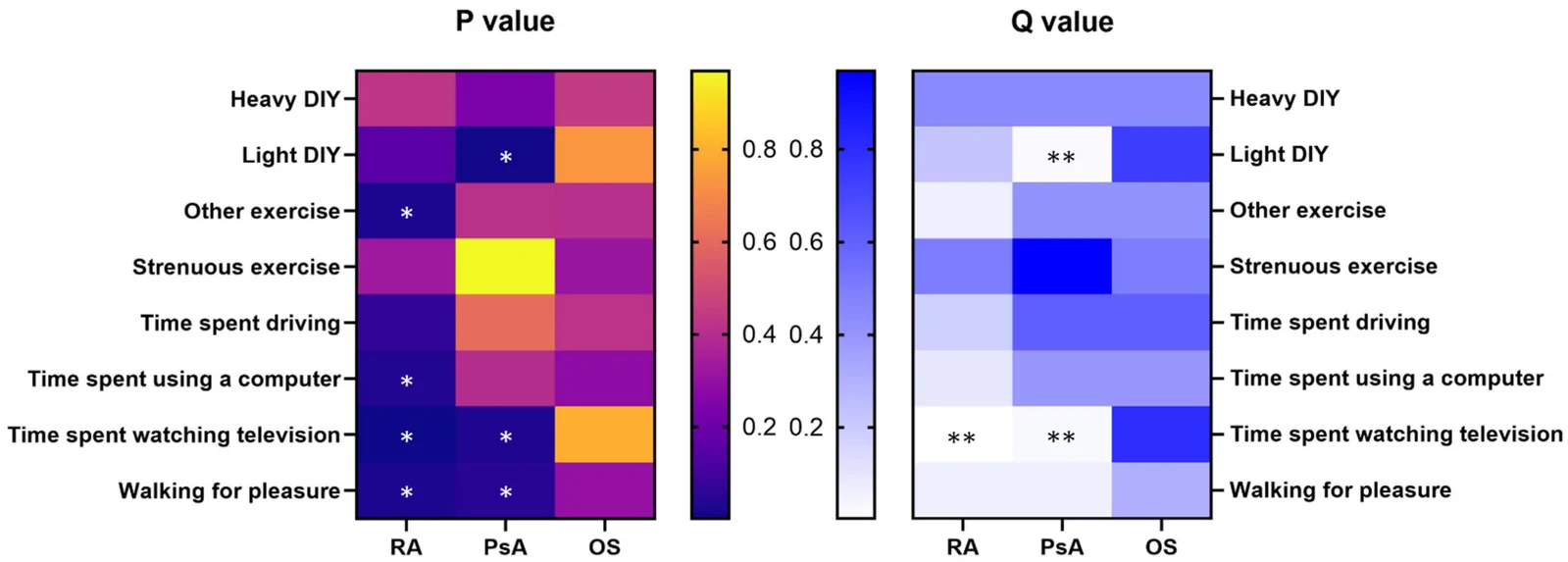
Heatmap summarizing the bidirectional Mendelian randomization (MR) results for physical activity/sedentary behavior (PA/SB) traits and osteoporosis (OS), rheumatoid arthritis (RA), and psoriatic arthritis (PsA). Nominal *p* values and false discovery rate (FDR)-adjusted Q values are shown. * and ** indicate statistical significance before and after FDR correction, respectively.

MR analyses examined the causal relationships of five PA traits and three SB traits with OS, RA, and PsA. Several exposure–outcome associations remained statistically significant after FDR correction. The overall MR estimates for OS, RA, and PsA are shown in Figure 2.

### 3.1. MR Estimates for Ra

After instrument selection, the numbers of SNPs retained as IVs were 11 for Light DIY, 18 for Heavy DIY, 6 for Strenuous sports, 20 for Walking for pleasure, 13 for Other exercises, 110 for Time spent watching television, 6 for Time spent driving, and 83 for Time spent using a computer.

**Figure 3:**
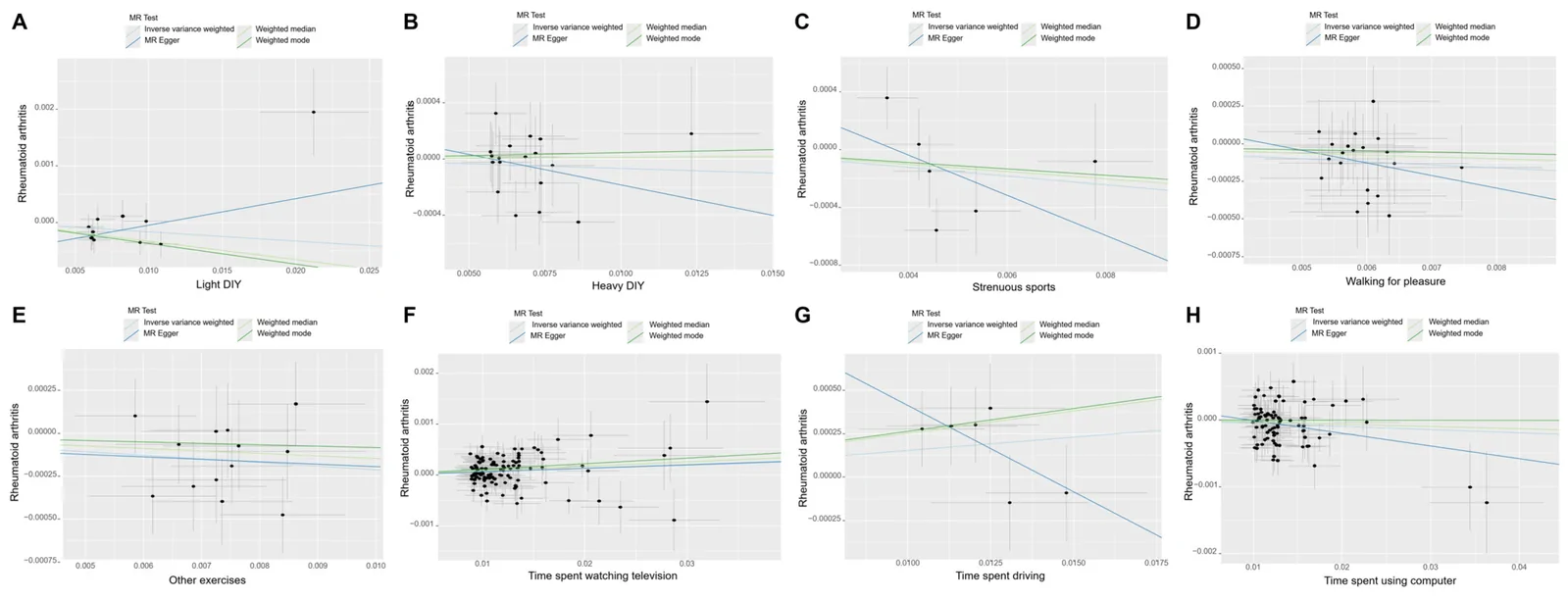
Scatter plots from Mendelian randomization (MR) analyses evaluating the relationships of physical activity (PA) and sedentary behavior (SB) traits with rheumatoid arthritis (RA). The panels correspond to the following trait–outcome pairs: (**A**) Light DIY and RA; (**B**) Heavy DIY and RA; (**C**) Strenuous sports and RA; (**D**) Walking for pleasure and RA; (**E**) Other exercises and RA; (**F**) Time spent watching television and RA; (**G**) Time spent driving and RA. (**H**) Time spent using a computer and RA. Black dots represent individual single-nucleotide polymorphisms (SNPs) used as genetic instruments.

MR analyses examined potential causal relationships of PA/SB traits with RA. The IVW method served as the primary analytical approach, with MR-Egger regression, weighted median, and weighted mode methods applied as complementary approaches. IVW analysis showed nominally significant associations for genetically predicted Walking for pleasure (*p* = 0.022, OR = 0.980, 95% CI = 0.963–0.997) (Figure 3D) and Other exercises (*p* = 0.021, OR = 0.979, 95% CI = 0.961–0.997) (Figure 3E). However, these associations were no longer statistically significant following FDR correction (FDR-adjusted *p* = 0.063 and 0.062, respectively). No nominally significant associations were observed for the remaining PA traits, including Light DIY (*p* = 0.150, OR = 0.984, 95% CI = 0.962–1.006) (Figure 3A), Heavy DIY (*p* = 0.424, OR = 0.993, 95% CI = 0.977–1.010) (Figure 3B), or Strenuous sports (*p* = 0.330, OR = 0.970, 95% CI = 0.912–1.031) (Figure 3C). Detailed MR estimates are provided in Supplementary Table S1.

Among the three SB traits, IVW analysis showed a significant association between genetically predicted Time spent watching television and RA (*p* = 0.002, OR = 1.006, 95% CI = 1.002–1.010) (Figure 3F), and this association retained statistical significance following FDR correction (FDR-adjusted *p* = 0.004). A nominally significant association was also observed for Time spent using a computer (*p* = 0.032, OR = 0.995, 95% CI = 0.991–1.000); however, this association was no longer statistically significant following FDR correction (FDR-adjusted *p* = 0.095). No significant association was observed for Time spent driving (*p* = 0.060, OR = 1.015, 95% CI = 0.999–1.032) (Figure 3G). All selected genetic instruments had F-statistics > 10 (Supplementary Tables S4–S8), supporting adequate instrument strength.

Sensitivity analyses were performed to evaluate the robustness of the MR findings. The MR-Egger intercept test showed no evidence of directional horizontal pleiotropy for any RA-associated traits (all *p* > 0.05) (Table 1). Similarly, the MR-PRESSO Global test detected no significant horizontal pleiotropy for any of the evaluated associations (all *p* > 0.05) (Table 1). No significant heterogeneity was detected by Cochran’s Q test across the evaluated RA exposure–outcome associations (all *p* > 0.05) (Table 2). Furthermore, leave-one-out analysis showed that removing each SNP in turn produced no substantial change in the causal estimates. This suggests that no single instrumental variable had a dominant influence on the results (Supplementary Figure S1).

**Figure 4:**
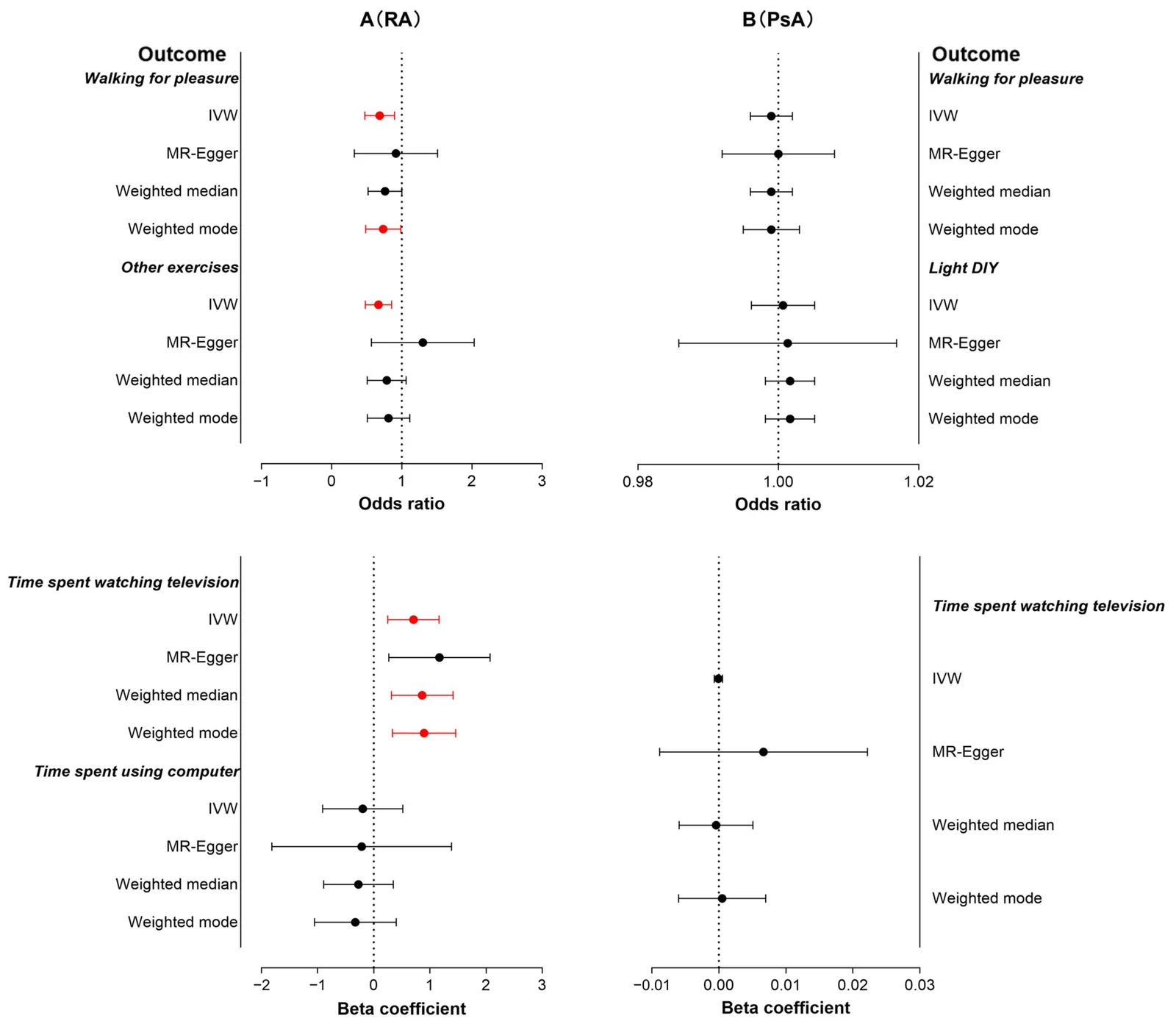
Reverse Mendelian randomization (MR) analyses of rheumatoid arthritis (RA) and psoriatic arthritis (PsA) with physical activity/sedentary behavior (PA/SB) traits. (**A**) Forest plots showing reverse MR estimates for RA; (**B**) Forest plots showing reverse MR estimates for PsA. Red points and error bars indicate statistically significant estimates (*p* < 0.05), whereas black points and error bars indicate non-significant estimates (*p* ≥ 0.05).

Subsequent reverse MR analysis examined the possibility of reverse causality. In the IVW analysis, genetic liability to RA was nominally associated with Walking for pleasure (OR = 0.662, 95% CI = 0.484–0.906, *p* = 0.010), Other exercises (OR = 0.651, 95% CI = 0.490–0.864, *p* = 0.003), and Time spent watching television (β = 0.708, 95% CI = 0.251–1.165, *p* = 0.002) (Figure 4A). All three associations retained statistical significance following FDR correction. The FDR-adjusted *p* values were 0.013 for Walking for pleasure, 0.006 for Other exercises, and 0.006 for Time spent watching television. For these associations, neither the MR-Egger intercept test nor the MR-PRESSO Global test detected evidence of horizontal pleiotropy (all *p* > 0.05). Cochran’s Q test also showed no significant heterogeneity across these associations (all *p* > 0.05). By contrast, no significant association was observed between genetic liability to RA and Time spent using a computer (β = −0.195, 95% CI = −0.908–0.517, *p* = 0.591; FDR-adjusted *p* = 0.591). However, significant heterogeneity was detected for this analysis by both the MR-Egger and IVW Q tests (Q-pval = 0.025 and Q-pval = 0.043, respectively). Supplementary Tables S9–S11 present the detailed reverse MR estimates and sensitivity analyses.

### 3.2. MR Estimates for Psa

After screening and harmonization, 10, 17, 6, 20, 13, 105, 6, and 76 SNPs were retained as IVs for Light DIY, Heavy DIY, Strenuous sports, Walking for pleasure, Other exercises, Time spent watching television, Time spent driving, and Time spent using a computer, respectively.

**Figure 5:**
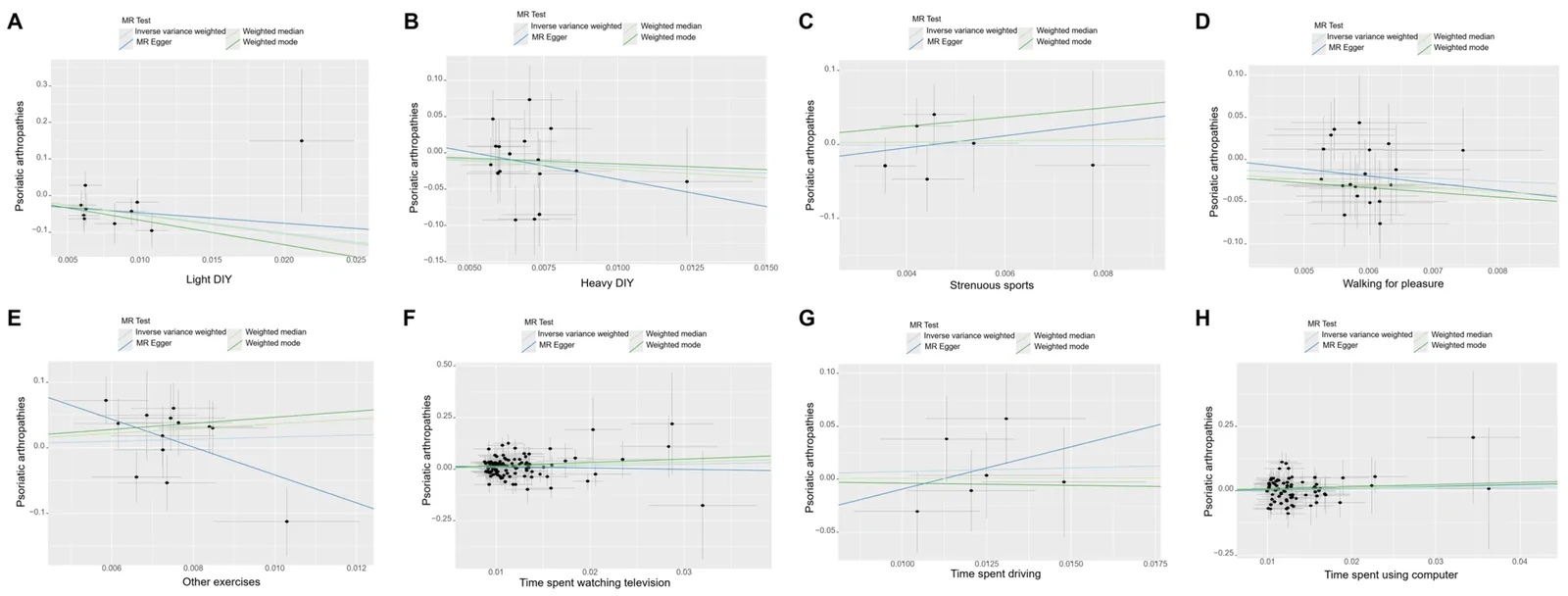
Scatter plots from Mendelian randomization (MR) analyses evaluating the relationships of physical activity (PA) and sedentary behavior (SB) traits with psoriatic arthritis (PsA). The panels correspond to the following trait–outcome pairs: (**A**) Light DIY and PsA; (**B**) Heavy DIY and PsA; (**C**) Strenuous sports and PsA; (**D**) Walking for pleasure and PsA; (**E**) Other exercises and PsA; (**F**) Time spent watching television and PsA; (**G**) Time spent driving and PsA. (**H**) Time spent using a computer and PsA. Black dots represent individual single-nucleotide polymorphisms (SNPs) used as genetic instruments.

MR analyses examined potential causal relationships of PA/SB traits with PsA. The IVW method was used as the primary analysis, with MR-Egger regression, weighted median, and weighted mode methods applied as complementary approaches. The IVW results indicated that genetically predicted Light DIY was significantly associated with PsA (*p* = 0.006, OR = 0.006, 95% CI = 0.0002–0.236) (Figure 5A). This association retained statistical significance following FDR correction (FDR-adjusted *p* = 0.018). Walking for pleasure showed a nominally significant association with PsA (*p* = 0.042, OR = 0.040, 95% CI = 0.002–0.887) (Figure 5D); however, statistical significance was lost after FDR correction (FDR-adjusted *p* = 0.063). No nominally significant associations were observed for the remaining PA/SB traits, including Heavy DIY (*p* = 0.237, OR = 0.150, 95% CI = 0.006–3.492) (Figure 5B), Strenuous sports (*p* = 0.969, OR = 0.846, 95% CI = 0.0002–4037.222) (Figure 5C), and Other exercises (*p* = 0.414, OR = 4.852, 95% CI = 0.110–214.562) (Figure 5E). Detailed MR estimates are provided in Supplementary Table S2.

Among the three SB traits, genetically predicted Time spent watching television was significantly associated with PsA (*p* = 0.028, OR = 2.205, 95% CI = 1.089–4.463) (Figure 5F), and the association retained statistical significance following FDR correction (FDR-adjusted *p* = 0.028). No significant associations were observed for Time spent driving (*p* = 0.607, OR = 2.044, 95% CI = 0.134–31.303) or Time spent using a computer (*p* = 0.397, OR = 1.396, 95% CI = 0.664–3.026) (Supplementary Table S2). All selected genetic instruments had F-statistics > 10 (Supplementary Tables S4–S8).

Sensitivity analyses were used to assess possible bias in the MR results. No evidence of directional horizontal pleiotropy was detected by the MR-Egger intercept test for PsA-related traits (all *p* > 0.05) (Table 1). Similarly, the MR-PRESSO Global test did not detect significant horizontal pleiotropy for the evaluated PsA exposure–outcome associations (all *p* > 0.05) (Table 1). Cochran’s Q test did not identify significant heterogeneity across the evaluated PsA exposure–outcome associations (all *p* > 0.05) (Table 2).

In the reverse MR analysis with genetic liability to PsA as the exposure, the IVW method showed no nominally significant associations with Light DIY (OR = 1.001, 95% CI = 0.996–1.005, *p* = 0.794), Walking for pleasure (OR = 0.999, 95% CI = 0.996–1.002, *p* = 0.453), or Time spent watching television (β = −0.0002, 95% CI = −0.006–0.006, *p* = 0.937) (Figure 4B). After FDR correction, none of these associations reached statistical significance. No directional horizontal pleiotropy was detected by the MR-Egger intercept test (all *p* > 0.05). MR-PRESSO results were unavailable because only three SNPs were included in each analysis. No significant heterogeneity was identified by Cochran’s Q test (all *p* > 0.05). Detailed reverse MR estimates and sensitivity analyses are provided in Supplementary Tables S9–S12.

### 3.3. Mr Estimates for Os

**Figure 6:**
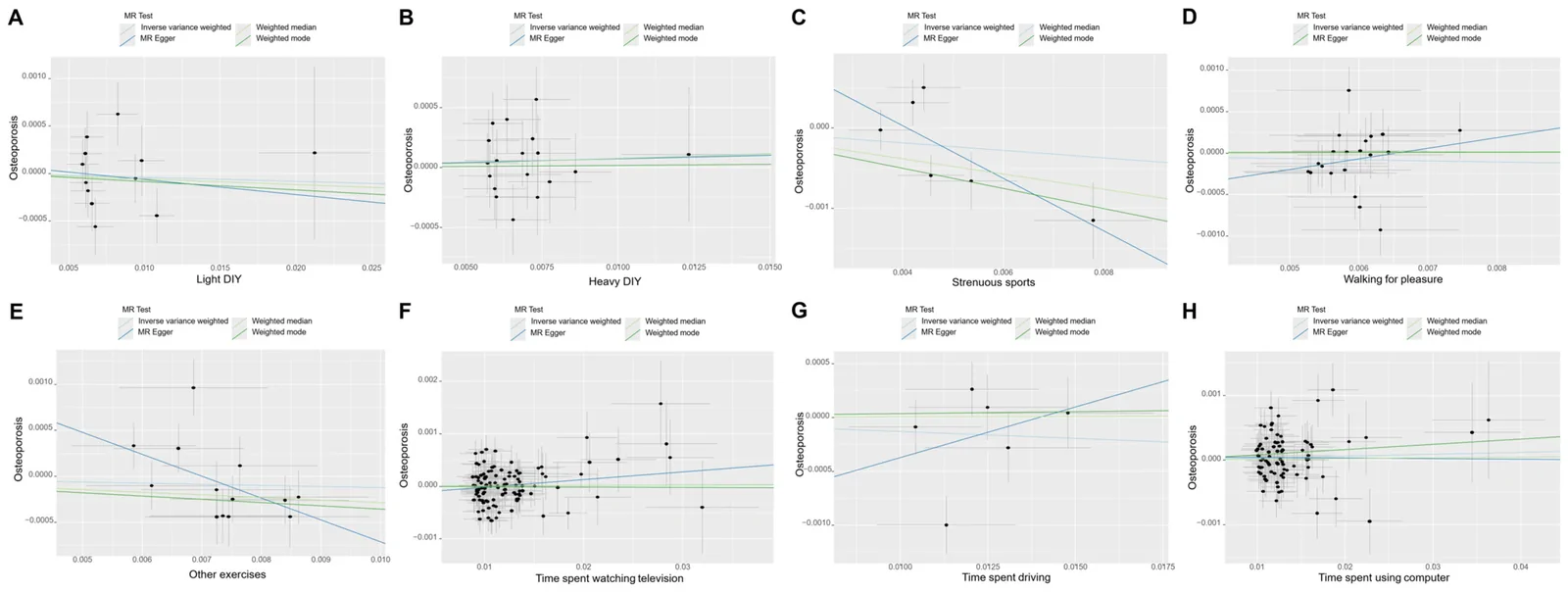
Scatter plots from Mendelian randomization (MR) analyses evaluating the relationships of physical activity (PA) and sedentary behavior (SB) traits with osteoporosis (OS). The panels correspond to the following trait–outcome pairs: (**A**) Light DIY and OS; (**B**) Heavy DIY and OS; (**C**) Strenuous sports and OS; (**D**) Walking for pleasure and OS; (**E**) Other exercises and OS; (**F**) Time spent watching television and OS; (**G**) Time spent driving and OS. (**H**) Time spent using a computer and OS. Black dots represent individual single-nucleotide polymorphisms (SNPs) used as genetic instruments.

None of the five PA traits or three SB traits showed a significant causal relationship with OS. The IVW estimates were as follows: Light DIY (*p* = 0.732, OR = 0.996, 95% CI = 0.972–1.020) (Figure 6A), Heavy DIY (*p* = 0.445, OR = 1.008, 95% CI = 0.988–1.028) (Figure 6B), Strenuous sports (*p* = 0.316, OR = 0.955, 95% CI = 0.872–1.045) (Figure 6C), Walking for pleasure (*p* = 0.300, OR = 0.986, 95% CI = 0.961–1.012) (Figure 6D), Other exercises (*p* = 0.409, OR = 0.988, 95% CI = 0.959–1.017) (Figure 6E), Time spent watching television (*p* = 0.799, OR = 1.001, 95% CI = 0.996–1.006) (Figure 6F), Time spent driving (*p* = 0.422, OR = 0.987, 95% CI = 0.957–1.019) (Figure 6G), and Time spent using a computer (*p* = 0.285, OR = 1.003, 95% CI = 0.998–1.009) (Figure 6H). Detailed MR results are summarized in Supplementary Table S3.

## 4. Discussion

To our knowledge, no previous bidirectional MR study has jointly investigated the relationships of IPAQ-derived domain-specific PA and SB traits with RA, PsA, and OS. In the forward MR analyses, genetically predicted liability to Light DIY was associated with lower odds of PsA after FDR correction. After FDR correction, longer genetically predicted television viewing time was linked to increased odds of both PsA and RA. Walking for pleasure showed a nominal inverse association with PsA, and computer use showed a nominal inverse association with RA. However, neither association remained significant after FDR correction. No evidence of associations between the examined PA/SB traits and OS was observed. In the reverse analyses, genetic liability to RA was linked to lower odds of Walking for pleasure and Other exercises and to longer television viewing time after FDR correction. No reverse associations were observed for PsA. The concordant forward and reverse findings for television viewing and RA may suggest a potential bidirectional relationship. However, these findings are best viewed as hypothesis-generating and warrant validation in independent studies. The effect estimates for Light DIY (OR = 0.006) and Walking for pleasure (OR = 0.040) were extreme in magnitude and should be interpreted cautiously. These estimates do not indicate that participation in these activities would directly reduce PsA risk to a near-zero level. The PA phenotypes were binary self-reported measures of participation during the previous 4 weeks. Therefore, the estimates reflect genetically predicted liability to participation on the original GWAS scale. They should not be interpreted as the effects of clinically defined PA frequency, duration, or intensity. The relatively small number of PsA cases and the wide CIs also indicate substantial uncertainty. In addition, the association for Walking for pleasure did not retain statistical significance following FDR correction. Therefore, greater emphasis should be placed on the direction and uncertainty of these estimates rather than their absolute magnitude. Replication in larger GWAS datasets and studies using objective PA measurements is needed.

Earlier research has examined the association between PA and PsA. According to a systematic review, regular PA may benefit patients with PsA by improving physical function, reducing pain, and enhancing quality of life. The review also suggested that aerobic exercise, resistance training, and flexibility exercises may contribute to symptom management [34]. A randomized controlled trial (*n* = 41) further demonstrated that resistance training significantly improved HAQ-S (*p* = 0.038) and BASDAI scores (*p* = 0.038), along with improvements in muscle strength, physical function, and quality of life among patients with PsA [11]. Although these studies support the clinical benefits of PA in patients with PsA, they do not establish whether specific PA domains influence the risk of developing PsA. In the present study, a bidirectional MR approach was applied to investigate potential genetically predicted relationships between domain-specific PA traits and PsA. Genetically predicted liability to Light DIY was associated with lower odds of PsA after FDR correction. Walking for pleasure was nominally inversely associated with PsA; however, statistical significance was not retained following FDR correction. These findings suggest that different PA domains may have distinct associations with PsA. However, the results should be interpreted cautiously. The sensitivity analyses did not provide evidence of horizontal pleiotropy, but the effect estimates were imprecise and the CIs were wide. The limited number of PsA cases and the use of self-reported binary PA phenotypes also increase uncertainty. Therefore, these findings are best regarded as hypothesis-generating and warrant confirmation in independent studies. Further research integrating genetic, molecular, and longitudinal behavioral data is required to clarify the mechanisms underlying these potential associations.

Numerous studies have reported beneficial associations between PA and functional outcomes in patients with RA [35,36]. These benefits include improved cardiovascular capacity, muscle strength, sleep quality, and reduced depressive symptoms [37,38]. Therefore, appropriate levels of PA may help improve quality of life and facilitate disease management and rehabilitation in individuals with RA. A Swedish observational study examined the association between regular PA performed five years before disease onset and subsequent RA severity. Among 617 patients with RA, those who reported regular PA before diagnosis had 42% lower odds of severe disease activity measured by DAS28 (OR = 0.58, 95% CI = 0.42–0.81), together with reduced risks of joint swelling and pain [39,40]. These findings suggest that pre-diagnosis PA patterns may be associated with milder clinical manifestations. In addition, the relationship between PA and RA may differ according to activity type, duration, and intensity, with longer-duration or moderate-to-high-intensity activities potentially showing greater clinical benefits [36,41]. However, residual confounding and reverse causation cannot be completely ruled out in observational studies. In our forward MR analysis, Walking for pleasure and Other exercises showed nominal inverse associations with RA, but neither association remained significant after FDR correction. Therefore, these results provide limited evidence that these PA traits influence RA risk. In the reverse MR analysis, genetic liability to RA was linked to lower odds of Walking for pleasure and Other exercises following FDR correction. This finding may indicate that RA liability influences PA patterns rather than providing strong evidence for bidirectional effects. Consistent with this interpretation, a previous review reported that individuals with RA generally have lower PA levels than healthy controls [42]. Reduced PA among patients with RA may result from multiple barriers, including patient-related factors such as limited motivation and knowledge, as well as healthcare-related factors such as insufficient emphasis on exercise prescription by rheumatologists [38,43]. These genetically predicted associations are best regarded as hypothesis-generating and warrant further validation.

In the present study, OS was defined as a broad binary self-reported diagnosis from UK Biobank field 20002. This endpoint did not distinguish BMD, fragility fracture, or OS at specific skeletal sites. No evidence was found for genetically predicted associations between the evaluated PA traits and this general diagnostic endpoint. This finding should not be taken to indicate that PA has no association with BMD, fracture risk, or site-specific OS. Previous observational and intervention studies have mainly focused on disease management, physical function, BMD, or fracture-related outcomes. For example, a randomized controlled trial involving patients with osteoporotic vertebral fractures found that a 12-month structured exercise intervention significantly enhanced health-related quality of life and balance performance [44]. Functional benefits of PA have also been reported in individuals with OS, particularly among older adults and women [12,45]. However, these outcomes differ from the broad binary diagnosis examined in our study. Domain-specific PA traits may affect BMD and fracture risk through different pathways. BMD responses depend on the type, intensity, and skeletal site of mechanical loading. Fracture risk also depends on factors such as balance, muscle strength, falls, and bone quality. A broad diagnostic endpoint may combine individuals with different skeletal sites, fracture histories, and clinical characteristics. This heterogeneity may reduce the ability to detect associations with specific PA domains. Low-impact activities, such as walking, may help maintain mobility and balance but may not provide sufficient mechanical loading to substantially increase BMD [46]. The PA traits examined in our study, including Walking for pleasure and Light DIY, may also fail to capture high-impact or resistance-based exercise. Therefore, our results indicate a lack of current genetic evidence for associations with a general self-reported OS diagnosis. They do not exclude potential associations with BMD, fragility fracture, or site-specific OS. Further MR studies using these more specific skeletal outcomes are needed.

In the present study, three SB domains were analyzed as exposures in relation to PsA, RA, and OS. In the forward MR analysis, longer genetically predicted television viewing time was linked to increased odds of both PsA and RA following FDR correction. The association with PsA was consistent with previous observational studies reporting relationships between SB and PsA risk [18,47]. Compared with other SB domains, television viewing involves very low energy expenditure and may be accompanied by prolonged sitting and passive energy intake. Prolonged sitting may also impair vascular function and contribute to chronic low-grade inflammation. This process may be related to elevated levels of C-reactive protein and pro-inflammatory cytokines, including TNF-α and IL-6, which are implicated in the pathogenesis of PsA [48,49,50]. However, these mechanisms were not directly assessed in the present study and should therefore be regarded only as possible explanations. In the reverse MR analysis, genetic liability to RA was also linked to longer television viewing time following FDR correction. The concordant findings in both directions may indicate a potential bidirectional association between television viewing and RA. However, this result should be considered hypothesis-generating and requires independent validation. By contrast, computer use showed only a nominal inverse association with RA in the forward MR analysis and did not remain significant after FDR correction. This finding should not be taken to indicate a protective effect of computer use. It may reflect broader socioeconomic or behavioral characteristics. Previous MR studies reported similar inverse associations and suggested that educational attainment may partly mediate this relationship [51,52]. The reverse analysis provided no evidence of an association between genetic liability to RA and computer use. No genetically predicted associations were observed between the examined SB domains and the broad OS diagnostic endpoint. Overall, the findings suggest that different SB domains may capture distinct genetic and behavioral characteristics. The observed associations require replication and further investigation before causal or clinical conclusions can be drawn.

This study has several strengths. First, to our knowledge, no previous bidirectional MR study has systematically examined potential genetically predicted associations between domain-specific PA/SB traits and OS, RA, and PsA. Compared with conventional observational studies, MR is less affected by environmental confounding and reverse causation because it uses genetic variants as IVs. However, MR cannot eliminate all potential sources of bias. Second, the robustness of the findings was evaluated using several complementary MR methods and sensitivity analyses. These analyses included heterogeneity tests, horizontal pleiotropy assessments, instrument-strength evaluation, and leave-one-out analysis. Third, the bidirectional design allowed us to examine whether genetic liability to the diseases might also influence PA or SB traits. This approach provided additional information on the possible direction of the observed associations. Finally, the use of publicly available GWAS summary statistics enabled the efficient investigation of multiple behavioral traits and disease outcomes. This design also avoided the substantial time and resources required for large prospective studies or randomized controlled trials. Nevertheless, the findings are best regarded as hypothesis-generating rather than conclusive evidence of causality.

Horizontal pleiotropy remains a potential limitation of the present study. The MR-Egger intercept and MR-PRESSO Global tests showed no evidence of significant horizontal pleiotropy for the evaluated associations. Leave-one-out analyses also indicated that no single SNP substantially influenced the main estimates. However, these sensitivity analyses cannot completely exclude pleiotropic effects. Some genetic instruments may still influence the outcomes through pathways unrelated to the investigated PA/SB traits. In addition, MR-PRESSO could not be performed for the reverse PsA analyses because only three SNPs were available. Therefore, the observed results are better regarded as potential genetically predicted associations rather than conclusive evidence of causality. Independent replication and further validation are required.

However, several limitations should be considered. Sex-stratified summary statistics were unavailable in the original GWAS datasets, preventing sex-specific MR analyses and leaving potential differences between men and women unclear. The PA and SB traits were derived from self-reported questionnaires rather than objective measurements, which may have introduced recall bias and exposure misclassification. Device-based assessments, such as accelerometry, provide more objective measurements but generally capture behavior over relatively short periods. Self-reported questionnaires may provide a better representation of habitual behaviors over extended periods and are widely used in large-scale genomic studies [53]. Nevertheless, the limitations of self-reported phenotypes cannot be excluded. The OS outcome was a broad binary self-reported diagnosis and did not distinguish BMD, fragility fracture, or osteoporosis at specific skeletal sites. Accordingly, the null results for OS should not be generalized to these more specific skeletal outcomes. The relatively limited number of PsA cases may have reduced statistical power and resulted in wide CIs for several estimates. Although the sensitivity analyses showed no strong evidence of horizontal pleiotropy for the evaluated associations, these methods cannot completely exclude pleiotropic effects or other violations of the MR assumptions. MR-PRESSO was also unavailable for the reverse PsA analyses because only three SNPs were included. Most participants were of European ancestry. This may restrict the applicability of the findings to populations of different ancestral backgrounds. Future studies using larger, ancestry-diverse datasets and more objective or clinically specific phenotypes are needed to validate these findings.

## 5. Conclusions

In summary, our bidirectional MR analyses identified several potential genetically predicted associations between domain-specific PA/SB traits and inflammatory musculoskeletal diseases. In the forward analyses, genetically predicted liability to Light DIY was associated with lower odds of PsA after FDR correction. Walking for pleasure showed a nominal inverse association with PsA, but statistical significance was not retained following FDR correction. Longer genetically predicted television viewing time was linked to increased odds of both PsA and RA following FDR correction. Computer use showed only a nominal inverse association with RA, and statistical significance was not retained following FDR correction. In the reverse analyses, genetic liability to RA was associated with lower odds of Walking for pleasure and Other exercises and with longer television viewing time. The concordant findings for television viewing and RA may suggest a potential bidirectional association, but this interpretation remains hypothesis-generating. No reverse associations were observed for PsA. We also found no evidence of associations between the examined PA/SB traits and the broad self-reported OS diagnosis. These null findings should not be generalized to BMD, fragility fracture, or site-specific OS. Overall, the findings require independent validation before causal or clinical conclusions can be drawn. Future research would benefit from larger and more diverse study populations, objective activity measurements, higher-intensity PA phenotypes, and more specific skeletal outcomes.

## Figures and Tables

**Table 1:** Pleiotropy tests for forward Mendelian randomization analyses of physical activity/sedentary behavior traits and rheumatoid arthritis or psoriatic arthritis.

Exposure	Outcome	MR Egger			MR-PRESSO Raw Causal *p*	MR-PRESSO Global Test *p*
		Intercept	SE	*p*		
Computer	RA	0.0002	0.0001	0.207	0.035	0.124
Driving	RA	0.001	0.001	0.199	0.101	0.541
Television	RA	−7.433 × 10^−6^	1.065 × 10^−4^	0.944	0.002	0.397
Other exercises	RA	−5.083 × 10^−5^	0.001	0.932	0.018	0.714
Light DIY	PsA	−0.017	0.054	0.760	0.011	0.725
Walking	PsA	0.031	0.124	0.803	0.021	0.871
Television	PsA	0.016	0.020	0.444	0.025	0.677

Abbreviations: Computer: Time spent using a computer; Driving: Time spent driving; Television: Time spent watching television; Walking: Walking for pleasure; RA: rheumatoid arthritis; PsA: psoriatic arthritis; MR: Mendelian randomization; MR-PRESSO: MR Pleiotropy Residual Sum and Outlier.

**Table 2:** Heterogeneity tests for forward Mendelian randomization analyses of physical activity/sedentary behavior traits and rheumatoid arthritis or psoriatic arthritis.

Exposure	Outcome	MR Egger			IVW		
		Q	Q-df	Q-pval	Q	Q-df	Q-pval
Computer	RA	94.964	81	0.138	96.860	82	0.125
Driving	RA	2.021	4	0.732	4.383	5	0.496
Television	RA	112.506	108	0.364	112.511	109	0.390
Other exercises	RA	8.662	11	0.653	8.670	12	0.731
Light DIY	PsA	6.444	8	0.598	6.544	9	0.684
Walking	PsA	12.370	18	0.828	12.434	19	0.866
Television	PsA	96.299	103	0.667	96.890	104	0.677

Abbreviations: Computer: Time spent using a computer; Driving: Time spent driving; Television: Time spent watching television; Walking: Walking for pleasure; RA: rheumatoid arthritis; PsA: psoriatic arthritis; MR: Mendelian randomization; IVW: inverse-variance weighted.

## Data Availability

The datasets generated during and/or analyzed during the current study are available from the corresponding author on reasonable request.

## Supplementary Materials

The following supporting information can be downloaded at: https://www.mdpi.com/article/10.3390/genes17080939/s1. Figure S1. Leave-one-out analyses for the associations between physical activity/sedentary behavior traits and rheumatoid arthritis, psoriatic arthritis, and osteoporosis. Table S1. Mendelian randomization estimates for associations between physical activity/sedentary behavior traits and rheumatoid arthritis. Table S2. Mendelian randomization estimates for associations between physical activity/sedentary behavior traits and psoriatic arthritis. Table S3. Mendelian randomization estimates for associations between physical activity/sedentary behavior traits and osteoporosis. Table S4. Genetic instrumental variables for Light DIY activity. Table S5. Genetic instrumental variables for Walking for pleasure. Table S6. Genetic instrumental variables for Other exercises. Table S7. Genetic instrumental variables for Time spent watching television. Table S8. Genetic instrumental variables for Time spent using a computer. Table S9. Pleiotropy test for reverse Mendelian randomization analysis of patients with rheumatoid arthritis and psoriatic arthritis with physical activity/sedentary behavior. Table S10. Heterogeneity test for reverse Mendelian randomization analysis of patients with rheumatoid arthritis and psoriatic arthritis with physical activity/sedentary behavior. Table S11. Reverse Mendelian randomization estimates between rheumatoid arthritis and different types of physical activity/sedentary behavior. Table S12. Reverse Mendelian randomization estimates between psoriatic arthritis and different types of physical activity/sedentary behavior. Table S13. Characteristics and phenotype definitions of GWAS datasets used for bidirectional Mendelian randomization analyses. Note: The osteoporosis and rheumatoid arthritis GWAS datasets from the IEU OpenGWAS database are identified by stable GWAS dataset identifiers (ebi-a-GCST90038656 and ebi-a-GCST90038685) rather than dataset-specific release identifiers. The ancestry information for these UK Biobank datasets was not reported in the GWAS Catalog (NR; country of recruitment: U.K.). The psoriatic arthropathies GWAS dataset was obtained from FinnGen Release 5 (endpoint: M13_PSORIARTH) and included participants of Finnish ancestry. Covariates adjusted in the original GWAS analyses included sex, age, BMI, assessment center, ethnicity, batch, and the first 20 genetic principal components (for osteoporosis and rheumatoid arthritis datasets); sex, age, genotyping batch, and genetic principal components according to the FinnGen GWAS pipeline (for the psoriatic arthropathies dataset).
