# Supplementary material for: Genetic Evidence for Causal Effects of Domain-Specific Physical Activity and Sedentary Behavior on Osteoporosis and Inflammatory Arthritis: A Bidirectional Mendelian Randomization Study
Source: Genes (Basel). 2026 Aug 12;17(8):939. doi: 10.3390/genes17080939 (PMC13511996; doi:10.3390/genes17080939)
Supplement: Supplementary file 1 [file genes-17-00939-s001.zip › genes-4462319-supplementary.pdf]

Table S1. Mendelian randomization estimates for associations between physical activity/sedentary behavior traits and rheumatoid arthritis.

Outcome	Rheumatoid arthritis					
Exposure	Method	OR	95% CI	P	FDR-adjusted P	SNPs
Light DIY	IVW	0.984	0.962-1.006	0.150	0.225	11
	MR-Egger	1.048	0.975-1.127	0.235	0.706	11
	Weighted median	0.968	0.943-0.993	0.012	0.035	11
	Weighted mode	0.964	0.943-0.993	0.057	0.114	11
Heavy DIY	IVW	0.993	0.977-1.010	0.424	0.445	18
	MR-Egger	0.958	0.860-1.067	0.444	0.712	18
	Weighted median	1.001	0.978-1.024	0.926	0.926	18
	Weighted mode	1.004	0.961-1.049	0.846	0.947	18
Strenuous sports	IVW	0.970	0.912-1.031	0.330	0.495	6
	MR-Egger	0.870	0.649-1.167	0.405	0.608	6
	Weighted median	0.975	0.915-1.039	0.437	0.655	6
	Weighted mode	0.978	0.887-1.079	0.674	0.674	6
Walking for pleasure	IVW	0.980	0.963-0.997	0.022	0.063	20
	MR-Egger	0.919	0.730-1.158	0.483	0.690	20
	Weighted median	0.988	0.965-1.011	0.287	0.431	20
	Weighted mode	0.992	0.949-1.037	0.726	0.955	20
Other exercises	IVW	0.979	0.961-0.997	0.021	0.062	13
	MR-Egger	0.986	0.843-1.153	0.860	0.860	13
	Weighted median	0.985	0.962-1.010	0.234	0.234	13
	Weighted mode	0.992	0.945-1.040	0.737	0.737	13
Time spent watching television	IVW	1.006	1.002-1.010	0.002	0.004	110
	MR-Egger	1.007	0.989-1.025	0.447	0.761	110
	Weighted median	1.009	1.003-1.015	0.004	0.008	110
	Weighted mode	1.011	0.996-1.027	0.166	0.298	110
Time spent driving	IVW	1.015	0.999-1.032	0.060	0.179	6
	MR-Egger	0.905	0.781-1.049	0.256	0.584	6
	Weighted median	1.026	1.005-1.046	0.013	0.039	6
	Weighted mode	1.027	0.994-1.061	0.176	0.525	6
Time spent using a computer	IVW	0.995	0.991-1.000	0.032	0.095	83
	MR-Egger	0.981	0.959-1.003	0.098	0.295	83
	Weighted median	0.997	0.990-1.003	0.298	0.553	83
	Weighted mode	1.000	0.985-1.015	0.997	0.997	83

Abbreviations: OR: odds ratio; CI: confidence interval; IVW: inverse-variance weighted; SNPs: single-nucleotide polymorphisms.

Table S2. Mendelian randomization estimates for associations between physical activity/sedentary behavior traits and psoriatic arthritis.

Outcome		Psoriatic arthropathies				
Exposure	Method	OR	95% CI	P	FDR-adjusted P	SNPs
Light DIY	IVW	0.006	0.0002-0.236	0.006	0.018	10
	MR-Egger	0.055	5.260×10^{-8}-5.718×10^{4}	0.692	0.748	10
	Weighted median	0.005	4.120×10^{-5}-0.694	0.035	0.053	10
	Weighted mode	0.001	1.830×10^{-6}-0.859	0.076	0.114	10
Heavy DIY	IVW	0.150	0.006-3.492	0.237	0.445	17
	MR-Egger	0.001	1.110×10^{-12}-2.762×10^{5}	0.474	0.712	17
	Weighted median	0.102	0.001-7.073	0.291	0.874	17
	Weighted mode	0.214	0.0001-380.625	0.692	0.947	17
Strenuous sports	IVW	0.846	0.0002-4037.222	0.969	0.969	6
	MR-Egger	3188.504	1.190×10^{-19}-8.530×10^{25}	0.775	0.775	6
	Weighted median	2.279	4.260×10^{-5}-1.220×10^{5}	0.882	0.882	6
	Weighted mode	492.765	4.390×10^{-5}-5.530×10^{9}	0.488	0.674	6
Walking for pleasure	IVW	0.040	0.002-0.887	0.042	0.063	20
	MR-Egger	$2.031\text{x}10^{-4}$	2.760×10^{-22}-1.490×10^{14}	0.690	0.690	20
	Weighted median	0.009	0.0001-0.581	0.027	0.081	20
	Weighted mode	0.004	1.700×10^{-6}-8.011	0.169	0.508	20
Other exercises	IVW	4.852	0.110-214.563	0.414	0.414	13
	MR-Egger	5.590×10^{-10}	1.710×10^{-20}-18.306	0.113	0.169	13
	Weighted median	37.552	0.410-3436.694	0.116	0.173	13
	Weighted mode	102.034	0.034-306296.293	0.280	0.419	13
Time spent watching television	IVW	2.205	1.089-4.463	0.028	0.028	105
	MR-Egger	0.582	0.018-18.653	0.761	0.761	105
	Weighted median	3.166	1.123-8.927	0.029	0.029	105
	Weighted mode	4.934	0.248-98.168	0.298	0.298	105
Time spent driving	IVW	2.044	0.134-31.303	0.607	0.607	6
	MR-Egger	2961.682	1.100×10^{-8}-7.960×10^{14}	0.584	0.584	6
	Weighted median	1.051	0.037-29.793	0.977	0.977	6
	Weighted mode	0.679	0.004-104.667	0.886	0.886	6
Time spent using a computer	IVW	1.396	0.644-3.026	0.397	0.397	76
	MR-Egger	2.041	0.026-159.721	0.749	0.944	76
	Weighted median	1.669	0.546-5.098	0.369	0.553	76
	Weighted mode	2.197	0.190-25.364	0.530	0.795	76

Abbreviations: OR: odds ratio; CI: confidence interval; IVW: inverse-variance weighted; SNPs: single-nucleotide polymorphisms.

Table S3. Mendelian randomization estimates for associations between physical activity/sedentary behavior traits and osteoporosis.

Outcome		Osteoporosis				
Exposure	Method	OR	95% CI	P	FDR-adjusted P	SNPs
Light DIY	IVW	0.996	0.972-1.020	0.732	0.732	12
	MR-Egger	0.984	0.896-1.081	0.748	0.748	12
	Weighted median	0.994	0.964-1.025	0.710	0.710	12
	Weighted mode	0.991	0.946-1.039	0.724	0.724	12
Heavy DIY	IVW	1.008	0.988-1.028	0.445	0.445	18
	MR-Egger	1.006	0.886-1.143	0.928	0.928	18
	Weighted median	1.008	0.981-1.035	0.588	0.882	18
	Weighted mode	1.002	0.951-1.055	0.947	0.947	18
Strenuous sports	IVW	0.955	0.872-1.045	0.316	0.495	6
	MR-Egger	0.720	0.498-1.042	0.157	0.470	6
	Weighted median	0.908	0.847-0.976	0.008	0.025	6
	Weighted mode	0.882	0.792-0.983	0.073	0.218	6
Walking for pleasure	IVW	0.986	0.961-1.012	0.300	0.300	20
	MR-Egger	1.135	0.800-1.612	0.486	0.690	20
	Weighted median	1.000	0.972-1.030	0.982	0.982	20
	Weighted mode	1.002	0.949-1.057	0.955	0.955	20
Other exercises	IVW	0.988	0.959-1.017	0.409	0.414	13
	MR-Egger	0.788	0.628-0.989	0.065	0.169	13
	Weighted median	0.972	0.944-1.000	0.052	0.156	13
	Weighted mode	0.965	0.926-1.006	0.118	0.355	13
Time spent watching television	IVW	1.001	0.996-1.006	0.799	0.799	111
	MR-Egger	1.015	0.992-1.038	0.205	0.205	111
	Weighted median	1.001	0.994-1.008	0.781	0.781	111
	Weighted mode	0.999	0.982-1.017	0.946	0.946	111
Time spent driving	IVW	0.987	0.957-1.019	0.422	0.607	6
	MR-Egger	1.098	0.807-1.495	0.583	0.584	6
	Weighted median	1.001	0.977-1.025	0.958	0.977	6
	Weighted mode	1.004	0.973-1.035	0.830	0.886	6
Time spent using a computer	IVW	1.003	0.998-1.009	0.285	0.397	83
	MR-Egger	0.999	0.971-1.027	0.944	0.944	83
	Weighted median	1.001	0.994-1.008	0.757	0.757	83
	Weighted mode	1.008	0.991-1.026	0.361	0.795	83

Abbreviations: OR: odds ratio; CI: confidence interval; IVW: inverse-variance weighted; SNPs: single-nucleotide polymorphisms.

Table S4. Genetic instrumental variables for Light DIY activity.

SNP	EA	OA	Beta	eaf	SE	P	R^2	F
Outcome: psoriatic arthritis								
rs10225528	G	C	-0.01082	0.734765	0.001174	3.10E-20	4.56E-05	84.94575
rs13413953	G	T	0.005917	0.359261	0.001082	4.60E-08	1.61E-05	29.88953
rs2120270	G	A	-0.00628	0.307991	0.001138	3.40E-08	1.68E-05	30.4772
rs2515938	T	C	0.006144	0.325333	0.001107	2.80E-08	1.66E-05	30.83077
rs28522006	G	A	-0.02123	0.020347	0.003663	6.90E-09	1.80E-05	33.57158
rs34713525	A	G	-0.0061	0.342223	0.001095	2.50E-08	1.67E-05	31.04437
rs35626515	A	C	-0.00941	0.4055	0.001056	5.20E-19	4.27E-05	79.36951
rs3791034	G	A	0.006216	0.33353	0.001102	1.7E-08	1.72E-05	31.81173
rs4848265	C	G	0.008239	0.176245	0.001368	1.7E-09	1.97E-05	36.2759
rs6751655	G	A	-0.00982	0.134615	0.00152	1E-10	2.25E-05	41.75048

Abbreviations: SNP: single-nucleotide polymorphism; EA: effect allele; OA: other allele; eaf: effect allele frequency; SE: standard error; R^2: proportion of variance in the exposure explained by the SNP; F: F-statistic indicating instrument strength.

Table S5. Genetic instrumental variables for Walking for pleasure.

SNP	EA	OA	Beta	eaf	SE	P	R^2	F
Outcome: rheumatoid arthritis								
rs11648192	T	C	0.006021	0.435784	0.000982	8.70E-10	1.78E-05	37.60441
rs11896330	A	G	-0.00594	0.6331	0.000976	1.10E-09	1.64E-05	37.08284
rs12042107	C	T	0.005302	0.54923	0.000943	1.90E-08	1.39E-05	31.62267
rs1827089	A	G	-0.00635	0.778923	0.001132	2.10E-08	1.39E-05	31.41716
rs1927902	C	T	0.006176	0.745501	0.001076	9.60E-09	1.45E-05	32.92989
rs1941943	T	C	0.006312	0.784349	0.001138	2.90E-08	1.35E-05	30.74375
rs2670012	T	C	0.005826	0.545137	0.000946	7.50E-10	1.68E-05	37.89483
rs2679059	T	A	0.005796	0.595739	0.000958	1.40E-09	1.62E-05	36.63212
rs2910829	A	G	0.005469	0.534643	0.000951	9.00E-09	1.49E-05	33.04491
rs3026380	C	G	0.005715	0.331196	0.00101	1.50E-08	1.45E-05	32.03214
rs34224566	T	C	-0.00617	0.26614	0.001058	5.50E-09	1.49E-05	34.00997
rs34898535	T	C	0.006015	0.377823	0.000966	4.70E-10	1.70E-05	38.80218
rs613872	T	G	-0.00746	0.825863	0.001235	1.50E-09	1.60E-05	36.5295
rs61955196	C	G	-0.0061	0.707856	0.001031	3.30E-09	1.54E-05	35.01611
rs6739738	T	C	-0.00563	0.493537	0.000937	1.90E-09	1.58E-05	36.10886
rs7133277	A	G	0.005415	0.64071	0.00098	3.30E-08	1.35E-05	30.50798
rs7207400	C	T	0.005856	0.271351	0.001052	2.60E-08	1.36E-05	30.98282
rs7610856	A	C	0.005599	0.429114	0.000949	3.70E-09	1.54E-05	34.79428
rs7617548	C	T	-0.00643	0.2084	0.001151	2.30E-08	1.36E-05	31.20556
rs968807	G	A	-0.00527	0.541059	0.000941	2.10E-08	1.38E-05	31.36852
Outcome: psoriatic arthritis								
rs11648192	T	C	0.006021	0.435784	0.000982	8.70E-10	1.78E-05	37.60441
rs11896330	A	G	-0.00594	0.6331	0.000976	1.10E-09	1.64E-05	37.08284
rs12042107	C	T	0.005302	0.54923	0.000943	1.90E-08	1.39E-05	31.62267
rs1827089	A	G	-0.00635	0.778923	0.001132	2.10E-08	1.39E-05	31.41716
rs1927902	C	T	0.006176	0.745501	0.001076	9.60E-09	1.45E-05	32.92989
rs1941943	T	C	0.006312	0.784349	0.001138	2.90E-08	1.35E-05	30.74375
rs2670012	T	C	0.005826	0.545137	0.000946	7.50E-10	1.68E-05	37.89483
rs2679059	T	A	0.005796	0.595739	0.000958	1.40E-09	1.62E-05	36.63212
rs2910829	A	G	0.005469	0.534643	0.000951	9.00E-09	1.49E-05	33.04491
rs3026380	C	G	0.005715	0.331196	0.00101	1.50E-08	1.45E-05	32.03214
rs34224566	T	C	-0.00617	0.26614	0.001058	5.50E-09	1.49E-05	34.00997
rs34898535	T	C	0.006015	0.377823	0.000966	4.70E-10	1.70E-05	38.80218
rs613872	T	G	-0.00746	0.825863	0.001235	1.50E-09	1.60E-05	36.5295
rs61955196	C	G	-0.0061	0.707856	0.001031	3.30E-09	1.54E-05	35.01611
rs6739738	T	C	-0.00563	0.493537	0.000937	1.90E-09	1.58E-05	36.10886
rs7133277	A	G	0.005415	0.64071	0.00098	3.30E-08	1.35E-05	30.50798
rs7207400	C	T	0.005856	0.271351	0.001052	2.60E-08	1.36E-05	30.98282
rs7610856	A	C	0.005599	0.429114	0.000949	3.70E-09	1.54E-05	34.79428
rs7617548	C	T	-0.00643	0.2084	0.001151	2.30E-08	1.36E-05	31.20556
rs968807	G	A	-0.00527	0.541059	0.000941	2.10E-08	1.38E-05	31.36852

Abbreviations: SNP: single-nucleotide polymorphism; EA: effect allele; OA: other allele; eaf: effect allele frequency; SE: standard error; R^2: proportion of variance in the exposure explained by the SNP; F: F-statistic indicating instrument strength.

Table S6. Genetic instrumental variables for Other exercises.

SNP	EA	OA	Beta	eaf	SE	P	R^2	F
Outcome: rheumatoid arthritis								
rs10135971	A	G	-0.00661	0.3386	0.001099	1.80E-09	1.96E-05	36.1654
rs11774212	T	C	0.005858	0.515802	0.001043	1.90E-08	1.71E-05	31.5469
rs11989077	A	G	0.00764	0.197083	0.001306	4.90E-09	1.85E-05	34.22962
rs12507369	C	A	0.00616	0.35025	0.001092	1.70E-08	1.73E-05	31.81217
rs12973258	C	T	-0.00745	0.183691	0.001344	2.90E-08	1.66E-05	30.74144
rs13022707	A	C	0.007353	0.23987	0.001219	1.60E-09	1.97E-05	36.36247
rs1691471	T	C	0.008398	0.376845	0.001074	5.40E-15	3.31E-05	61.09314
rs169413	A	G	-0.00862	0.258842	0.001186	3.60E-13	2.85E-05	52.85589
rs55773130	C	T	0.008482	0.182249	0.001346	3.00E-10	2.14E-05	39.68795
rs62062137	A	G	0.006861	0.224664	0.001246	3.70E-08	1.64E-05	30.3154
rs72948529	C	A	-0.00725	0.213968	0.001273	1.20E-08	1.77E-05	32.44204
rs7615206	C	T	-0.00752	0.429927	0.001049	7.70E-13	2.77E-05	51.36348
rs9835772	T	A	0.007252	0.243634	0.001211	2.10E-09	1.94E-05	35.88866

Abbreviations: SNP: single-nucleotide polymorphism; EA: effect allele; OA: other allele; eaf: effect allele frequency; SE: standard error; R^2: proportion of variance in the exposure explained by the SNP; F: F-statistic indicating instrument strength.

Table S7. Genetic instrumental variables for Time spent watching television.

SNP	EA	OA	Beta	eaf	SE	P	R^2	F
				Outcome: rheumatoid arthritis				
rs10109061	G	A	-0.00897	0.443183	0.001619	3.10E-08	3.97E-05	30.67228
rs10189857	G	A	0.014686	0.432324	0.001605	5.70E-20	0.000106	83.71824
rs10269099	T	G	0.009149	0.391234	0.001632	2.10E-08	3.99E-05	31.41191
rs10739499	G	C	-0.00924	0.662566	0.001685	4.20E-08	3.81E-05	30.04955
rs10765777	C	A	-0.0113	0.391753	0.001636	4.90E-12	6.09E-05	47.73019
rs111901094	T	G	0.012711	0.182164	0.002098	1.40E-09	4.81E-05	36.72028
rs11191129	T	C	-0.00985	0.422818	0.001609	9.30E-10	4.73E-05	37.4663
rs11222919	G	T	-0.01221	0.175374	0.002102	6.30E-09	4.31E-05	33.74983
rs11245482	C	T	0.010189	0.385493	0.001636	4.80E-10	4.92E-05	38.77208
rs114600294	C	G	0.010821	0.20804	0.001955	3.10E-08	3.86E-05	30.62531
rs114755463	A	G	0.013259	0.16775	0.002148	6.70E-10	4.91E-05	38.09656
rs115608101	T	C	-0.01354	0.130399	0.002371	1.10E-08	4.16E-05	32.59875
rs11662211	T	C	-0.00945	0.505628	0.001591	2.90E-09	4.46E-05	35.24255
rs11680095	T	C	-0.00919	0.592713	0.001634	1.90E-08	4.08E-05	31.61916
rs11696187	T	C	-0.01357	0.160229	0.002171	4.20E-10	4.95E-05	39.02933
rs11700249	G	T	0.009433	0.408795	0.001621	5.90E-09	4.30E-05	33.86023
rs11714337	A	G	-0.00991	0.430094	0.001611	7.60E-10	4.82E-05	37.85503
rs11877758	G	T	0.011075	0.312562	0.001724	1.30E-10	5.27E-05	41.28487
rs11911112	C	A	-0.01074	0.365887	0.001653	8.00E-11	5.36E-05	42.2476
rs12045585	A	G	-0.01506	0.131234	0.00241	4.10E-10	5.17E-05	39.0684
rs12214364	G	T	0.009046	0.415458	0.001645	3.80E-08	3.97E-05	30.23402
rs12553324	G	C	-0.01356	0.415158	0.001615	4.50E-17	8.94E-05	70.5236
rs1291871	C	T	0.009162	0.514085	0.001598	9.90E-09	4.19E-05	32.86168
rs13014947	A	G	0.010607	0.574722	0.001624	6.50E-11	5.50E-05	42.66295
rs13107325	T	C	0.02032	0.075099	0.003018	1.70E-11	5.74E-05	45.32657
rs1324491	A	G	0.013413	0.130982	0.002357	1.30E-08	4.10E-05	32.36974
rs1727332	T	C	0.013151	0.753551	0.001849	1.10E-12	6.42E-05	50.60123
rs17789218	C	T	-0.01092	0.243949	0.001849	3.60E-09	4.40E-05	34.84526
rs178203	C	T	0.012014	0.749551	0.001835	5.90E-11	5.42E-05	42.84495
rs180396	T	C	0.01005	0.746657	0.001835	4.30E-08	3.82E-05	30.00743
rs1826510	A	G	-0.01273	0.828498	0.002264	1.90E-08	4.61E-05	31.62104
rs184332798	A	G	-0.02777	0.026113	0.005003	2.80E-08	3.92E-05	30.81494
rs1889996	G	T	0.011968	0.738965	0.001811	3.90E-11	5.53E-05	43.66437
rs1993092	C	T	-0.00952	0.389657	0.001631	5.40E-09	4.31E-05	34.0514
rs2073869	T	C	-0.0138	0.166613	0.002139	1.10E-10	5.29E-05	41.61059
rs2106164	C	T	-0.00939	0.531731	0.001598	4.20E-09	4.39E-05	34.51327
rs2185490	C	A	0.009373	0.617654	0.001645	1.20E-08	4.15E-05	32.4708
rs2240857	G	T	0.015931	0.141014	0.002302	4.50E-12	6.15E-05	47.89037
rs2283	G	A	-0.00927	0.339277	0.001676	3.20E-08	3.85E-05	30.59187
rs2332818	C	T	0.009884	0.731272	0.001799	4.00E-08	3.84E-05	30.17223
rs2352984	C	T	0.019757	0.430943	0.001604	7.40E-35	0.000191	151.7031
rs2479968	G	A	0.020589	0.05112	0.003702	2.70E-08	4.11E-05	30.92238
rs249960	G	A	-0.0118	0.182119	0.002066	1.10E-08	4.15E-05	32.63276
rs262890	G	A	0.013239	0.298989	0.001738	2.60E-14	7.35E-05	58.0422
rs263771	A	C	0.012014	0.233452	0.001885	1.90E-10	5.17E-05	40.60192

rs2646351	A	G	0.008914	0.452916	0.001598	2.40E-08	3.94E-05	31.10149
rs2678662	G	T	0.011146	0.608337	0.001632	8.60E-12	5.92E-05	46.62083
rs2725371	G	A	-0.01326	0.695652	0.001735	2.10E-14	7.45E-05	58.47891
rs2906604	C	T	0.010401	0.497337	0.001586	5.50E-11	5.41E-05	42.99394
rs3138499	C	A	0.010964	0.518183	0.001605	8.50E-12	6.00E-05	46.65829
rs34094119	G	A	0.009082	0.525005	0.001596	1.30E-08	4.11E-05	32.38191
rs34811474	A	G	-0.01316	0.230691	0.001887	3.10E-12	6.14E-05	48.59846
rs35797019	G	A	-0.00921	0.392335	0.001628	1.50E-08	4.05E-05	32.03995
rs362312	C	T	-0.00952	0.424168	0.001612	3.50E-09	4.43E-05	34.89259
rs3754970	C	T	0.009702	0.502922	0.001599	1.30E-09	4.71E-05	36.82831
rs3810496	C	T	0.009371	0.61619	0.001644	1.20E-08	4.15E-05	32.48264
rs4076457	T	C	-0.01004	0.256946	0.001824	3.70E-08	3.85E-05	30.29861
rs4110177	A	G	0.009427	0.367281	0.001657	1.30E-08	4.13E-05	32.37491
rs4303732	C	T	-0.01073	0.40162	0.001621	3.70E-11	5.53E-05	43.77946
rs4339469	G	T	0.012529	0.62921	0.001647	2.80E-14	7.32E-05	57.86078
rs4469687	G	A	0.008799	0.483936	0.00159	3.10E-08	3.87E-05	30.6209
rs4567133	A	C	-0.01302	0.810356	0.002038	1.70E-10	5.21E-05	40.82154
rs4747438	T	C	-0.01182	0.676512	0.001703	3.90E-12	6.11E-05	48.15249
rs4788616	G	T	-0.01018	0.391065	0.001631	4.30E-10	4.94E-05	38.98178
rs4847408	C	G	-0.0104	0.649373	0.001663	4.10E-10	4.92E-05	39.06814
rs494566	T	C	0.010166	0.332086	0.001696	2.00E-09	4.58E-05	35.92984
rs57555420	T	C	0.010175	0.281686	0.001772	9.40E-09	4.19E-05	32.95692
rs58541850	A	G	0.02139	0.058526	0.003387	2.70E-10	5.04E-05	39.87179
rs6102912	C	T	-0.01091	0.41003	0.001615	1.40E-11	5.76E-05	45.60277
rs6125907	A	C	0.016182	0.092033	0.00276	4.50E-09	4.38E-05	34.38148
rs61743199	G	A	0.017384	0.071839	0.003079	1.60E-08	4.03E-05	31.87378
rs61864793	C	T	-0.01114	0.251695	0.001834	1.20E-09	4.68E-05	36.89429
rs62145951	C	T	-0.01203	0.263053	0.001804	2.60E-11	5.61E-05	44.43287
rs62199883	A	C	0.013843	0.48612	0.00159	3.20E-18	9.57E-05	75.7684
rs6493583	G	C	0.013362	0.137725	0.002328	9.50E-09	4.24E-05	32.93649
rs6511708	C	T	-0.01208	0.664796	0.001685	7.50E-13	6.51E-05	51.4104
rs68056254	T	G	0.013495	0.150874	0.002225	1.30E-09	4.67E-05	36.79187
rs6814554	A	G	0.013528	0.47436	0.001596	2.40E-17	9.13E-05	71.82265
rs6850494	C	A	0.00966	0.386016	0.001634	3.40E-09	4.42E-05	34.93805
rs6895658	C	T	-0.01298	0.193239	0.002013	1.10E-10	5.25E-05	41.58331
rs6994132	C	T	-0.00996	0.579236	0.001611	6.20E-10	4.84E-05	38.25
rs7089973	A	C	0.009295	0.380267	0.001646	1.60E-08	4.07E-05	31.89182
rs71658797	A	T	0.015758	0.121135	0.00244	1.10E-10	5.29E-05	41.69646
rs7184800	A	G	-0.01345	0.302842	0.00173	7.40E-15	7.64E-05	60.47605
rs72673939	C	G	0.0121	0.183051	0.002058	4.10E-09	4.38E-05	34.55727
rs73571431	T	C	0.015838	0.109769	0.002553	5.50E-10	4.90E-05	38.49557
rs73946726	A	C	0.031959	0.02024	0.005708	2.20E-08	4.05E-05	31.34919
rs749056	G	T	-0.01002	0.304306	0.001732	7.20E-09	4.25E-05	33.48163
rs749671	A	G	-0.01108	0.371805	0.001645	1.60E-11	5.74E-05	45.38783
rs7539775	A	G	0.009969	0.743419	0.00182	4.30E-08	3.79E-05	30.00513
rs75499503	T	C	-0.01843	0.219683	0.001948	3.10E-21	0.000116	89.46348
rs75641275	C	A	0.015433	0.14333	0.00227	1.00E-11	5.85E-05	46.23892
rs7708324	G	A	-0.00989	0.377232	0.001639	1.60E-09	4.59E-05	36.4041
rs77273138	A	T	0.023477	0.045412	0.00384	9.70E-10	4.78E-05	37.37918

rs7798292	A	G	-0.00972	0.43452	0.001603	1.30E-09	4.64E-05	36.76417
rs78227853	T	C	-0.02836	0.024787	0.005139	3.40E-08	3.89E-05	30.45264
rs7899206	G	T	-0.00978	0.492382	0.00161	1.30E-09	4.78E-05	36.85235
rs7921305	A	G	-0.01147	0.252545	0.001831	3.70E-10	4.97E-05	39.25695
rs79373894	C	T	-0.02873	0.034386	0.00444	9.80E-11	5.48E-05	41.86217
rs801733	C	A	-0.01249	0.357688	0.001659	5.00E-14	7.17E-05	56.72324
rs814197	G	T	-0.01068	0.466816	0.001594	2.00E-11	5.68E-05	44.94817
rs872169	G	C	0.011346	0.205676	0.001968	8.20E-09	4.21E-05	33.23313
rs898751	T	C	0.010021	0.493393	0.001591	3.00E-10	5.02E-05	39.66448
rs9300594	G	A	0.011202	0.253582	0.001829	9.10E-10	4.75E-05	37.50458
rs9471333	T	C	-0.01043	0.552259	0.001598	6.70E-11	5.38E-05	42.59206
rs9834970	C	T	-0.00891	0.497804	0.001587	2.00E-08	3.97E-05	31.49091
rs9867121	A	C	-0.0117	0.184147	0.00206	1.40E-08	4.11E-05	32.23946
rs9867437	C	A	-0.0103	0.460371	0.001602	1.30E-10	5.28E-05	41.36609
rs9880023	T	G	0.009647	0.556427	0.001608	2.00E-09	4.59E-05	35.99396
rs996234	A	G	-0.01054	0.516342	0.001648	1.60E-10	5.55E-05	40.91593
Outcome: psoriatic arthritis								
rs10109061	G	A	-0.00897	0.443183	0.001619	3.10E-08	3.97E-05	30.67228
rs10189857	G	A	0.014686	0.432324	0.001605	5.70E-20	0.000106	83.71824
rs10269099	T	G	0.009149	0.391234	0.001632	2.10E-08	3.99E-05	31.41191
rs10739499	G	C	-0.00924	0.662566	0.001685	4.20E-08	3.81E-05	30.04955
rs10765777	C	A	-0.0113	0.391753	0.001636	4.90E-12	6.09E-05	47.73019
rs11191129	T	C	-0.00985	0.422818	0.001609	9.30E-10	4.73E-05	37.4663
rs11222919	G	T	-0.01221	0.175374	0.002102	6.30E-09	4.31E-05	33.74983
rs11245482	C	T	0.010189	0.385493	0.001636	4.80E-10	4.92E-05	38.77208
rs114600294	C	G	0.010821	0.20804	0.001955	3.10E-08	3.86E-05	30.62531
rs114755463	A	G	0.013259	0.16775	0.002148	6.70E-10	4.91E-05	38.09656
rs115608101	T	C	-0.01354	0.130399	0.002371	1.10E-08	4.16E-05	32.59875
rs11662211	T	C	-0.00945	0.505628	0.001591	2.90E-09	4.46E-05	35.24255
rs11680095	T	C	-0.00919	0.592713	0.001634	1.90E-08	4.08E-05	31.61916
rs11696187	T	C	-0.01357	0.160229	0.002171	4.20E-10	4.95E-05	39.02933
rs11700249	G	T	0.009433	0.408795	0.001621	5.90E-09	4.30E-05	33.86023
rs11714337	A	G	-0.00991	0.430094	0.001611	7.60E-10	4.82E-05	37.85503
rs11877758	G	T	0.011075	0.312562	0.001724	1.30E-10	5.27E-05	41.28487
rs11911112	C	A	-0.01074	0.365887	0.001653	8.00E-11	5.36E-05	42.2476
rs12045585	A	G	-0.01506	0.131234	0.00241	4.10E-10	5.17E-05	39.0684
rs12214364	G	T	0.009046	0.415458	0.001645	3.80E-08	3.97E-05	30.23402
rs1291871	C	T	0.009162	0.514085	0.001598	9.90E-09	4.19E-05	32.86168
rs13014947	A	G	0.010607	0.574722	0.001624	6.50E-11	5.50E-05	42.66295
rs13107325	T	C	0.02032	0.075099	0.003018	1.70E-11	5.74E-05	45.32657
rs1324491	A	G	0.013413	0.130982	0.002357	1.30E-08	4.10E-05	32.36974
rs1727332	T	C	0.013151	0.753551	0.001849	1.10E-12	6.42E-05	50.60123
rs17789218	C	T	-0.01092	0.243949	0.001849	3.60E-09	4.40E-05	34.84526
rs178203	C	T	0.012014	0.749551	0.001835	5.90E-11	5.42E-05	42.84495
rs180396	T	C	0.01005	0.746657	0.001835	4.30E-08	3.82E-05	30.00743
rs1826510	A	G	-0.01273	0.828498	0.002264	1.90E-08	4.61E-05	31.62104
rs1889996	G	T	0.011968	0.738965	0.001811	3.90E-11	5.53E-05	43.66437
rs1993092	C	T	-0.00952	0.389657	0.001631	5.40E-09	4.31E-05	34.0514
rs2073869	T	C	-0.0138	0.166613	0.002139	1.10E-10	5.29E-05	41.61059

rs2106164	C	T	-0.00939	0.531731	0.001598	4.20E-09	4.39E-05	34.51327
rs2185490	C	A	0.009373	0.617654	0.001645	1.20E-08	4.15E-05	32.4708
rs2240857	G	T	0.015931	0.141014	0.002302	4.50E-12	6.15E-05	47.89037
rs2283	G	A	-0.00927	0.339277	0.001676	3.20E-08	3.85E-05	30.59187
rs2332818	C	T	0.009884	0.731272	0.001799	4.00E-08	3.84E-05	30.17223
rs2352984	C	T	0.019757	0.430943	0.001604	7.40E-35	0.000191	151.7031
rs2479968	G	A	0.020589	0.05112	0.003702	2.70E-08	4.11E-05	30.92238
rs249960	G	A	-0.0118	0.182119	0.002066	1.10E-08	4.15E-05	32.63276
rs262890	G	A	0.013239	0.298989	0.001738	2.60E-14	7.35E-05	58.0422
rs263771	A	C	0.012014	0.233452	0.001885	1.90E-10	5.17E-05	40.60192
rs2646351	A	G	0.008914	0.452916	0.001598	2.40E-08	3.94E-05	31.10149
rs2678662	G	T	0.011146	0.608337	0.001632	8.60E-12	5.92E-05	46.62083
rs2725371	G	A	-0.01326	0.695652	0.001735	2.10E-14	7.45E-05	58.47891
rs2857693	T	G	0.010658	0.365548	0.001646	9.60E-11	5.27E-05	41.90662
rs2906604	C	T	0.010401	0.497337	0.001586	5.50E-11	5.41E-05	42.99394
rs3138499	C	A	0.010964	0.518183	0.001605	8.50E-12	6.00E-05	46.65829
rs34094119	G	A	0.009082	0.525005	0.001596	1.30E-08	4.11E-05	32.38191
rs34811474	A	G	-0.01316	0.230691	0.001887	3.10E-12	6.14E-05	48.59846
rs35797019	G	A	-0.00921	0.392335	0.001628	1.50E-08	4.05E-05	32.03995
rs362312	C	T	-0.00952	0.424168	0.001612	3.50E-09	4.43E-05	34.89259
rs3754970	C	T	0.009702	0.502922	0.001599	1.30E-09	4.71E-05	36.82831
rs3810496	C	T	0.009371	0.61619	0.001644	1.20E-08	4.15E-05	32.48264
rs4076457	T	C	-0.01004	0.256946	0.001824	3.70E-08	3.85E-05	30.29861
rs4110177	A	G	0.009427	0.367281	0.001657	1.30E-08	4.13E-05	32.37491
rs4303732	C	T	-0.01073	0.40162	0.001621	3.70E-11	5.53E-05	43.77946
rs4339469	G	T	0.012529	0.62921	0.001647	2.80E-14	7.32E-05	57.86078
rs4469687	G	A	0.008799	0.483936	0.00159	3.10E-08	3.87E-05	30.6209
rs4567133	A	C	-0.01302	0.810356	0.002038	1.70E-10	5.21E-05	40.82154
rs4747438	T	C	-0.01182	0.676512	0.001703	3.90E-12	6.11E-05	48.15249
rs4788616	G	T	-0.01018	0.391065	0.001631	4.30E-10	4.94E-05	38.98178
rs4847408	C	G	-0.0104	0.649373	0.001663	4.10E-10	4.92E-05	39.06814
rs494566	T	C	0.010166	0.332086	0.001696	2.00E-09	4.58E-05	35.92984
rs57555420	T	C	0.010175	0.281686	0.001772	9.40E-09	4.19E-05	32.95692
rs6102912	C	T	-0.01091	0.41003	0.001615	1.40E-11	5.76E-05	45.60277
rs61743199	G	A	0.017384	0.071839	0.003079	1.60E-08	4.03E-05	31.87378
rs61864793	C	T	-0.01114	0.251695	0.001834	1.20E-09	4.68E-05	36.89429
rs62145951	C	T	-0.01203	0.263053	0.001804	2.60E-11	5.61E-05	44.43287
rs62199883	A	C	0.013843	0.48612	0.00159	3.20E-18	9.57E-05	75.7684
rs6493583	G	C	0.013362	0.137725	0.002328	9.50E-09	4.24E-05	32.93649
rs6511708	C	T	-0.01208	0.664796	0.001685	7.50E-13	6.51E-05	51.4104
rs68056254	T	G	0.013495	0.150874	0.002225	1.30E-09	4.67E-05	36.79187
rs6814554	A	G	0.013528	0.47436	0.001596	2.40E-17	9.13E-05	71.82265
rs6850494	C	A	0.00966	0.386016	0.001634	3.40E-09	4.42E-05	34.93805
rs6895658	C	T	-0.01298	0.193239	0.002013	1.10E-10	5.25E-05	41.58331
rs6994132	C	T	-0.00996	0.579236	0.001611	6.20E-10	4.84E-05	38.25
rs7089973	A	C	0.009295	0.380267	0.001646	1.60E-08	4.07E-05	31.89182
rs71658797	A	T	0.015758	0.121135	0.00244	1.10E-10	5.29E-05	41.69646
rs7184800	A	G	-0.01345	0.302842	0.00173	7.40E-15	7.64E-05	60.47605
rs72673939	C	G	0.0121	0.183051	0.002058	4.10E-09	4.38E-05	34.55727

rs73571431	T	C	0.015838	0.109769	0.002553	5.50E-10	4.90E-05	38.49557
rs73946726	A	C	0.031959	0.02024	0.005708	2.20E-08	4.05E-05	31.34919
rs749056	G	T	-0.01002	0.304306	0.001732	7.20E-09	4.25E-05	33.48163
rs749671	A	G	-0.01108	0.371805	0.001645	1.60E-11	5.74E-05	45.38783
rs7539775	A	G	0.009969	0.743419	0.00182	4.30E-08	3.79E-05	30.00513
rs75499503	T	C	-0.01843	0.219683	0.001948	3.10E-21	0.000116	89.46348
rs75641275	C	A	0.015433	0.14333	0.00227	1.00E-11	5.85E-05	46.23892
rs77273138	A	T	0.023477	0.045412	0.00384	9.70E-10	4.78E-05	37.37918
rs7798292	A	G	-0.00972	0.43452	0.001603	1.30E-09	4.64E-05	36.76417
rs78227853	T	C	-0.02836	0.024787	0.005139	3.40E-08	3.89E-05	30.45264
rs7899206	G	T	-0.00978	0.492382	0.00161	1.30E-09	4.78E-05	36.85235
rs7921305	A	G	-0.01147	0.252545	0.001831	3.70E-10	4.97E-05	39.25695
rs79373894	C	T	-0.02873	0.034386	0.00444	9.80E-11	5.48E-05	41.86217
rs801733	C	A	-0.01249	0.357688	0.001659	5.00E-14	7.17E-05	56.72324
rs814197	G	T	-0.01068	0.466816	0.001594	2.00E-11	5.68E-05	44.94817
rs872169	G	C	0.011346	0.205676	0.001968	8.20E-09	4.21E-05	33.23313
rs898751	T	C	0.010021	0.493393	0.001591	3.00E-10	5.02E-05	39.66448
rs9300594	G	A	0.011202	0.253582	0.001829	9.10E-10	4.75E-05	37.50458
rs9471333	T	C	-0.01043	0.552259	0.001598	6.70E-11	5.38E-05	42.59206
rs9834970	C	T	-0.00891	0.497804	0.001587	2.00E-08	3.97E-05	31.49091
rs9867121	A	C	-0.0117	0.184147	0.00206	1.40E-08	4.11E-05	32.23946
rs9867437	C	A	-0.0103	0.460371	0.001602	1.30E-10	5.28E-05	41.36609
rs9880023	T	G	0.009647	0.556427	0.001608	2.00E-09	4.59E-05	35.99396
rs996234	A	G	-0.01054	0.516342	0.001648	1.60E-10	5.55E-05	40.91593

Abbreviations: SNP: single-nucleotide polymorphism; EA: effect allele; OA: other allele; eaf: effect allele frequency; SE: standard error; R^2: proportion of variance in the exposure explained by the SNP; F: F-statistic indicating instrument strength.

Table S8. Genetic instrumental variables for Time spent using a computer.

SNP	EA	OA	Beta	eaf	SE	P	R^2	F
				Outcome: rheumatoid arthritis				
rs10208088	T	C	-0.01024	0.579637	0.001849	3.00E-08	5.11E-05	30.68537
rs1037091	T	C	-0.01548	0.327191	0.001983	5.90E-15	0.000105	60.91812
rs10518019	G	A	0.009944	0.476252	0.001823	4.90E-08	4.93E-05	29.73799
rs10828248	G	A	0.010653	0.344788	0.001918	2.80E-08	5.13E-05	30.84631
rs11259902	A	C	0.012908	0.200416	0.002286	1.60E-08	5.34E-05	31.89512
rs112600282	G	A	-0.01691	0.110458	0.002923	7.20E-09	5.62E-05	33.48136
rs113851275	A	G	0.018611	0.107713	0.002943	2.50E-10	6.66E-05	39.99395
rs11634155	C	T	-0.01161	0.335422	0.001948	2.50E-09	6.01E-05	35.55579
rs11652437	A	C	0.012784	0.323689	0.001978	1.00E-10	7.15E-05	41.77254
rs117405403	C	G	0.011624	0.584606	0.001848	3.20E-10	6.56E-05	39.55704
rs11749912	G	A	-0.01288	0.574515	0.001855	3.70E-12	8.11E-05	48.25616
rs11766392	T	G	-0.01274	0.284846	0.002024	3.10E-10	6.61E-05	39.58462
rs11942953	C	T	-0.01046	0.536706	0.001839	1.30E-08	5.44E-05	32.32401
rs12128707	G	A	0.012036	0.263894	0.002079	7.10E-09	5.63E-05	33.51754
rs12145677	A	G	0.015578	0.297237	0.001999	6.40E-15	0.000101	60.76214
rs1229984	C	T	-0.03445	0.97263	0.005522	4.40E-10	6.32E-05	38.9107
rs12521638	G	A	0.010077	0.447056	0.001838	4.20E-08	5.02E-05	30.05579
rs12553324	G	C	0.017475	0.415898	0.001852	3.90E-21	0.000148	89.02638
rs12706626	A	G	0.010409	0.383718	0.001877	2.90E-08	5.12E-05	30.7618
rs12820967	C	T	0.011892	0.322363	0.001967	1.50E-09	6.18E-05	36.55661
rs12946454	T	A	-0.01224	0.268016	0.002065	3.10E-09	5.88E-05	35.12351
rs13262595	G	A	0.015632	0.560611	0.001837	1.80E-17	0.00012	72.37065
rs13422733	T	C	-0.01515	0.125533	0.002761	4.10E-08	5.04E-05	30.11499
rs136553	T	C	0.011311	0.376531	0.001885	2.00E-09	6.01E-05	35.99414
rs1395020	A	G	-0.011	0.302632	0.001993	3.40E-08	5.11E-05	30.46566
rs1448355	T	C	0.012261	0.618488	0.001886	8.00E-11	7.09E-05	42.26268
rs1469249	A	G	-0.01297	0.210879	0.002248	8.00E-09	5.60E-05	33.2767
rs147543875	T	C	-0.03632	0.02267	0.006422	1.50E-08	5.85E-05	31.99315
rs1648906	A	G	-0.01104	0.321177	0.001969	2.10E-08	5.32E-05	31.4283
rs166835	T	C	-0.01082	0.556188	0.001843	4.30E-09	5.78E-05	34.48102
rs16912540	G	A	-0.01582	0.136623	0.002661	2.70E-09	5.91E-05	35.36278
rs17167210	A	G	-0.0114	0.435554	0.001841	6.00E-10	6.39E-05	38.30653
rs17789218	C	T	0.012695	0.245186	0.00212	2.10E-09	5.97E-05	35.87171
rs17862355	G	T	-0.01121	0.442114	0.001837	1.00E-09	6.20E-05	37.24284
rs1987942	C	T	-0.01106	0.616935	0.001895	5.40E-09	5.78E-05	34.04666
rs1999244	T	A	0.013066	0.220345	0.002206	3.20E-09	5.87E-05	35.06505
rs2032780	C	T	0.01235	0.405991	0.001875	4.50E-11	7.36E-05	43.37398
rs2041687	G	T	0.011709	0.549172	0.001869	3.80E-10	6.79E-05	39.23042
rs2068625	C	T	0.014175	0.698468	0.001989	1.00E-12	8.46E-05	50.77582
rs206965	C	T	-0.01294	0.791153	0.002243	7.90E-09	5.54E-05	33.29667
rs2120461	T	C	0.012184	0.661091	0.001926	2.50E-10	6.65E-05	40.00261
rs2220599	G	C	0.012313	0.36947	0.001897	8.50E-11	7.06E-05	42.1403
rs246723	G	A	-0.01067	0.590096	0.001871	1.20E-08	5.50E-05	32.50236
rs2588543	T	C	0.010908	0.67203	0.00195	2.20E-08	5.25E-05	31.28674
rs2734833	A	G	0.012375	0.606682	0.001871	3.80E-11	7.31E-05	43.72567

rs2748985	C	T	0.012497	0.544439	0.001834	9.40E-12	7.75E-05	46.44722
rs2756121	A	T	-0.01156	0.367201	0.001901	1.20E-09	6.21E-05	37.00188
rs2761438	G	A	-0.01133	0.623567	0.001882	1.70E-09	6.03E-05	36.27281
rs28710456	C	T	-0.01041	0.49014	0.001827	1.20E-08	5.41E-05	32.42607
rs306755	C	T	0.010372	0.47539	0.001828	1.40E-08	5.37E-05	32.17852
rs34238696	G	A	-0.01682	0.106778	0.00296	1.30E-08	5.39E-05	32.26836
rs3730399	G	A	-0.02279	0.064399	0.003721	9.10E-10	6.26E-05	37.50732
rs422115	T	A	0.012261	0.250875	0.002112	6.40E-09	5.65E-05	33.69809
rs4704043	T	C	0.011608	0.713608	0.002018	8.90E-09	5.51E-05	33.07519
rs4852252	C	T	0.010193	0.563856	0.001839	3.00E-08	5.11E-05	30.71843
rs56229818	C	T	-0.01012	0.484413	0.001826	3.00E-08	5.12E-05	30.71542
rs58638214	T	C	-0.01283	0.397608	0.001869	6.70E-12	7.89E-05	47.11739
rs6028090	A	G	0.012534	0.55468	0.001851	1.30E-11	7.76E-05	45.87003
rs613872	T	G	-0.01571	0.826663	0.002415	7.70E-11	7.07E-05	42.32958
rs6449708	C	T	-0.01058	0.531571	0.001833	7.90E-09	5.57E-05	33.30223
rs6780848	G	T	0.011368	0.269946	0.002057	3.30E-08	5.09E-05	30.54167
rs6935828	T	C	0.010167	0.556131	0.001836	3.10E-08	5.10E-05	30.64998
rs7020477	G	A	-0.01197	0.266981	0.002067	7.00E-09	5.61E-05	33.54797
rs707926	A	G	0.014537	0.150465	0.002548	1.20E-08	5.40E-05	32.54366
rs7209653	C	T	-0.0134	0.294886	0.002002	2.20E-11	7.46E-05	44.77185
rs7281293	C	A	0.012627	0.247862	0.002129	3.00E-09	5.94E-05	35.18931
rs72828532	C	T	0.015684	0.179279	0.002382	4.50E-11	7.24E-05	43.36738
rs72847500	C	T	0.015805	0.121024	0.002811	1.90E-08	5.31E-05	31.62324
rs7288455	G	A	-0.01055	0.566933	0.001846	1.10E-08	5.46E-05	32.64687
rs73578186	T	C	-0.01229	0.323945	0.001962	3.70E-10	6.62E-05	39.26453
rs7526112	G	T	-0.01085	0.361752	0.001898	1.10E-08	5.43E-05	32.65323
rs75550998	T	G	-0.02239	0.053578	0.004095	4.60E-08	5.08E-05	29.89314
rs7564844	A	G	-0.01234	0.700653	0.001991	5.60E-10	6.39E-05	38.45278
rs76112266	G	C	-0.01626	0.166004	0.002455	3.50E-11	7.32E-05	43.86229
rs7630869	T	C	0.016113	0.304231	0.00198	4.00E-16	0.00011	66.2491
rs76824303	C	A	-0.02044	0.099986	0.003121	5.80E-11	7.52E-05	42.89134
rs7904398	T	C	-0.0102	0.503287	0.001828	2.40E-08	5.20E-05	31.13555
rs7968738	A	G	-0.01242	0.263717	0.00208	2.40E-09	5.99E-05	35.65886
rs79720045	C	T	-0.01276	0.400744	0.001898	1.80E-11	7.82E-05	45.23061
rs806795	A	G	0.010356	0.470383	0.001824	1.40E-08	5.34E-05	32.23027
rs8102851	C	T	0.012967	0.217206	0.002244	7.60E-09	5.72E-05	33.38297
rs9375188	T	C	0.015852	0.483548	0.001832	5.00E-18	0.000126	74.89627
rs9537571	A	G	0.018954	0.095569	0.003107	1.10E-09	6.21E-05	37.22007

Abbreviations: SNP: single-nucleotide polymorphism; EA: effect allele; OA: other allele; eaf: effect allele frequency; SE: standard error; R^2: proportion of variance in the exposure explained by the SNP; F: F-statistic indicating instrument strength.

Table S9. Pleiotropy test for reverse Mendelian randomization analysis of patients with rheumatoid arthritis and psoriatic arthritis with physical activity/sedentary behavior.

Exposure	Outcome	MR Egger			MR-PRESSO raw causal P	MR-PRESSO Global Test P
		Intercept	SE	P		
RA	Walking	-0.001	0.001	0.591	0.037	0.231
RA	Other exercises	-0.002	0.001	0.069	0.021	0.388
RA	Television	-0.002	0.002	0.296	0.022	0.552
RA	Computer	7.734×10^{-5}	0.003	0.979	0.608	0.080
PsA	Walking	-0.001	0.002	0.806	0.020	NA
PsA	Light DIY	-0.0004	0.004	0.933	0.010	NA
PsA	Television	-0.004	0.004	0.526	0.024	NA

Abbreviations: Computer: Time spent using a computer; Walking: Walking for pleasure; Television: Time spent watching television; RA: rheumatoid arthritis; PsA: psoriatic arthritis; MR: Mendelian randomization; MR PRESSO: MR Pleiotropy Residual Sum and Outlier.

Table S10. Heterogeneity test for reverse Mendelian randomization analysis of patients with rheumatoid arthritis and psoriatic arthritis with physical activity/sedentary behavior.

Exposure	Outcome	MR Egger			IVW		
		Q	Q-df	Q-pval	Q	Q-df	Q-pval
RA	Walking	10.095	6	0.121	10.637	7	0.155
RA	Other exercises	2.157	6	0.905	7.041	7	0.425
RA	Television	4.476	5	0.483	5.835	6	0.442
RA	Computer	14.465	6	0.025	14.467	7	0.043
PsA	Walking	0.391	1	0.532	0.490	2	0.783
PsA	Light DIY	3.362	1	0.067	3.400	2	0.183
PsA	Television	1.457	1	0.227	2.693	2	0.260

Abbreviations: Computer: Time spent using a computer; Walking: Walking for pleasure; Television: Time spent watching television; RA: rheumatoid arthritis; PsA: psoriatic arthritis; MR: Mendelian randomization, IVW: inverse-variance weighted.

Table S11. Reverse Mendelian randomization estimates between rheumatoid arthritis and different types of physical activity/sedentary behavior.

Exposure	Rheumatoid arthritis					
Outcome	Method	OR	95% CI	P	FDR-adjusted P	SNPs
Walking for pleasure	IVW	0.662	0.484-0.906	0.010	0.013	8
	MR-Egger	0.788	0.397-1.564	0.522	0.801	8
	Weighted median	0.735	0.534-1.012	0.059	0.118	8
	Weighted mode	0.707	0.500-0.999	0.090	0.180	8
Other exercises	IVW	0.651	0.490-0.864	0.003	0.006	13
	MR-Egger	1.162	0.646-2.090	0.634	0.801	13
	Weighted median	0.753	0.526-1.077	0.121	0.161	13
	Weighted mode	0.774	0.530-1.131	0.227	0.303	13
Outcome	Method	β	95% CI	P	FDR-adjusted P	SNPs
Time spent watching television	IVW	0.708	0.251-1.165	0.002	0.006	7
	MR-Egger	1.170	0.268-2.073	0.052	0.208	7
	Weighted median	0.864	0.316-1.413	0.002	0.008	7
	Weighted mode	0.897	0.335-1.460	0.020	0.080	7
Time spent using a computer	IVW	-0.195	-0.908-0.517	0.591	0.591	8
	MR-Egger	-0.215	-1.814-1.385	0.801	0.801	8
	Weighted median	-0.271	-0.891-0.349	0.392	0.392	8
	Weighted mode	-0.327	-1.054-0.401	0.408	0.408	8

Abbreviations: OR: odds ratio; CI: confidence interval; IVW: inverse-variance weighted; SNPs: single-nucleotide polymorphisms; β: beta coefficient.

Table S12. Reverse Mendelian randomization estimates between psoriatic arthritis and different types of physical activity/sedentary behavior.

Exposure	Psoriatic arthritis					
Outcome	Method	OR	95% CI	P	FDR-adjusted P	SNPs
Walking for pleasure	IVW	0.999	0.996-1.002	0.453	0.937	3
	MR-Egger	1.000	0.992-1.008	0.992	0.992	3
	Weighted median	0.999	0.996-1.002	0.581	0.872	3
	Weighted mode	0.999	0.995-1.003	0.705	0.897	3
Light DIY	IVW	1.001	0.996-1.005	0.794	0.937	3
	MR-Egger	1.001	0.986-1.017	0.892	0.992	3
	Weighted median	1.002	0.998-1.005	0.414	0.872	3
	Weighted mode	1.002	0.998-1.005	0.488	0.897	3
Outcome	Method	β	95% CI	P	FDR-adjusted P	SNPs
Time spent watching television	IVW	-0.0002	-0.006-0.006	0.937	0.937	3
	MR-Egger	0.007	-0.009-0.022	0.565	0.992	3
	Weighted median	-0.0002	-0.006-0.005	0.932	0.932	3
	Weighted mode	0.0005	-0.006-0.007	0.897	0.897	3

Abbreviations: OR: odds ratio; CI: confidence interval; IVW: inverse-variance weighted; SNPs: single-nucleotide polymorphisms; β: beta coefficient.

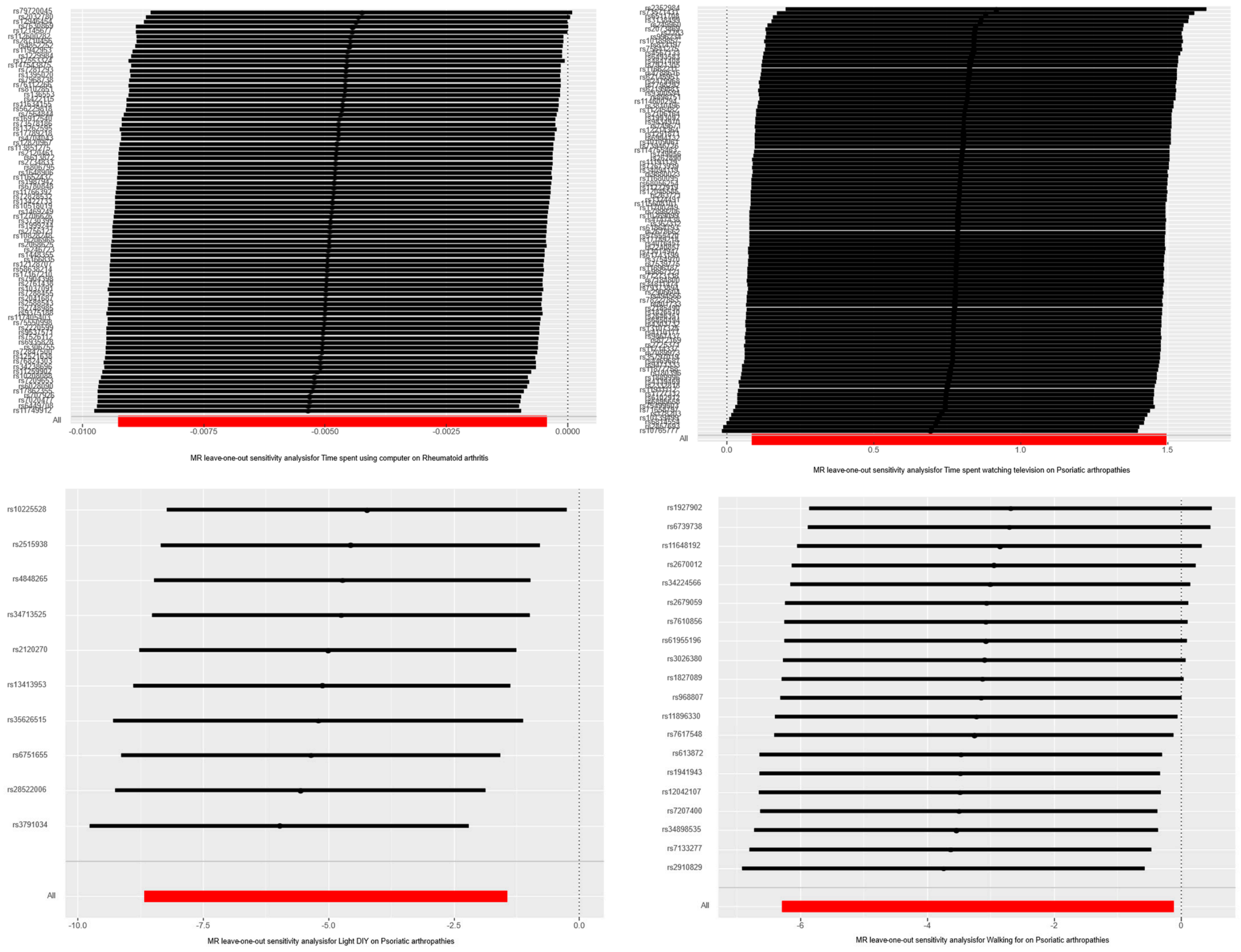

Figure S1. Leave-one-out analyses for the associations between physical activity/sedentary behavior traits and rheumatoid arthritis, psoriatic arthritis, and osteoporosis.

Table S13. Characteristics and phenotype definitions of GWAS datasets used for bidirectional Mendelian randomization analyses.

Trait	Role in MR analysis	Accession number/GWAS dataset ID	Phenotype definition and diagnostic criteria/coding
Light DIY	Exposure	ukb-b-11495	See Methods, Section 2.2.
Heavy DIY	Exposure	ukb-b-13184	See Methods, Section 2.2.
Strenuous sports	Exposure	ukb-b-7663	See Methods, Section 2.2.
Walking for pleasure	Exposure	ukb-b-7337	See Methods, Section 2.2.
Other exercises	Exposure	ukb-b-8764	See Methods, Section 2.2.
Time spent watching television	Exposure	ukb-b-5192	See Methods, Section 2.2.
Time spent driving	Exposure	ukb-b-3793	See Methods, Section 2.2.
Time spent using a computer	Exposure	ukb-b-4522	See Methods, Section 2.2.
Light DIY	Outcome	ukb-b-11495	See Methods, Section 2.2.
Walking for pleasure	Outcome	ukb-b-7337	See Methods, Section 2.2.
Other exercises	Outcome	ukb-b-8764	See Methods, Section 2.2.
Time spent watching television	Outcome	ukb-b-5192	See Methods, Section 2.2.
Time spent using a computer	Outcome	ukb-b-4522	See Methods, Section 2.2.
Rheumatoid arthritis	Exposure	ebi-a-GCST90038685	Self-reported rheumatoid arthritis diagnosis from UK Biobank (field 20002)
Psoriatic arthropathies	Exposure	finn-b-M13_PSORIARTH	FinnGen Release 5 endpoint M13_PSORIARTH (psoriatic arthropathies)
Osteoporosis	Exposure	ebi-a-GCST90038656	Self-reported osteoporosis diagnosis from UK Biobank (field 20002)
Rheumatoid arthritis	Outcome	ebi-a-GCST90038685	Self-reported rheumatoid arthritis diagnosis from UK Biobank (field 20002)
Psoriatic arthropathies	Outcome	finn-b-M13_PSORIARTH	FinnGen Release 5 endpoint M13_PSORIARTH (psoriatic arthropathies)
Osteoporosis	Outcome	ebi-a-GCST90038656	Self-reported osteoporosis diagnosis from UK Biobank (field 20002)

Note: The osteoporosis and rheumatoid arthritis GWAS datasets from the IEU OpenGWAS database are identified by stable GWAS dataset identifiers (ebi-a-GCST90038656 and ebi-a-GCST90038685) rather than dataset-specific release identifiers. The ancestry information for these UK Biobank datasets was not reported in the GWAS Catalog (NR; country of recruitment: U.K.). The psoriatic arthropathies GWAS dataset was obtained from FinnGen Release 5 (endpoint: M13_PSORIARTH) and included participants of Finnish ancestry. Covariates adjusted in the original GWAS analyses included sex, age, BMI, assessment center, ethnicity, batch, and the first 20 genetic principal components (for osteoporosis and rheumatoid arthritis datasets); sex, age, genotyping batch, and genetic principal components according to the FinnGen GWAS pipeline (for the psoriatic arthropathies dataset).
